# scMGCL: accurate and efficient integration representation of single-cell multi-omics data

**DOI:** 10.1093/bioinformatics/btaf392

**Published:** 2025-07-09

**Authors:** Zhenglong Cheng, Risheng Lu, Shixiong Zhang

**Affiliations:** School of Computer Science and Technology, Xidian University, Xi’an, Shaanxi 710126, China; School of Computer Science and Technology, Xidian University, Xi’an, Shaanxi 710126, China; School of Computer Science and Technology, Xidian University, Xi’an, Shaanxi 710126, China

## Abstract

**Motivation:**

Single-cell multi-omics data integration is essential for understanding cellular states and disease mechanisms, yet integrating heterogeneous data modalities remains a challenge. We present scMGCL, a graph contrastive learning framework for robust integration of single-cell ATAC-seq and RNA-seq data. Our approach leverages self-supervised learning on cell–cell similarity graphs, in which each modality’s graph structure serves as an augmentation for the other. This cross-modality contrastive paradigm enables the learning of biologically meaningful, shared representations while preserving modality-specific features.

**Results:**

Benchmarking against state-of-the-art methods demonstrates that scMGCL outperforms others in cell-type clustering, label transfer accuracy, and preservation of marker-gene correlations. Additionally, scMGCL significantly improves computational efficiency, reducing runtime and memory usage. The method’s effectiveness is further validated through extensive analyses of cell-type similarity and functional consistency, providing a powerful tool for multi-omics data exploration.

**Availability and implementation:**

Code and datasets are released at https://github.com/zlCreator/scMGCL.

## 1 Introduction

Single-cell sequencing technologies have revolutionized our ability to dissect cellular heterogeneity at unprecedented resolution. While single-cell RNA sequencing (scRNA-seq) profiles the transcriptomes and single-cell ATAC sequencing (scATAC-seq) maps the chromatin accessibility landscapes, these single-modality approaches provide only partial insights into cellular states and functions (Ashuach *et al.* 2023). This limitation fundamentally constrains our understanding of complex cellular behaviors and gene regulatory mechanisms. Recent advances in single-cell multi-omics technologies enable simultaneous profiling of multiple molecular layers within the same individual cells, offering new opportunities for comprehensive cellular characterization.

Current computational methods for integrating single-cell multi-omics data face several significant challenges. Traditional approaches relying on linear dimensionality reduction techniques, including latent Dirichlet allocation (LDA), principal component analysis (PCA), and multidimensional scaling (MDS), often fail to capture the complex, nonlinear relationships inherent in single-cell data (Gong *et al.* 2021). Methods like iCluster (Shen *et al.* 2009) that employ linear models for integration struggle similarly with the high dimensionality and sparsity characteristic of scRNA-seq and scATAC-seq data, as well as the inherent biological heterogeneity of cellular populations.

More advanced integration strategies have attempted to address these limitations through various approaches. Seurat (Satija *et al.* 2015) and Harmony (Korsunsky *et al.* 2019) improve alignment by incorporating cell–cell relationship graphs, but their memory requirements are prohibitive for large datasets. MultiMAP (Jain *et al.* 2021), based on UMAP’s nonlinear embedding, often converges to local optima due to local optima and lacks consideration of modality resolution differences. SCALEX (Xiong *et al.* 2022) and uniPort (Cao *et al.* 2022) employ sophisticated nonlinear mappings, but their computational costs scale poorly with dataset size. Batch correction methods, such as scCorrector (Guo *et al.* 2024), face a tradeoff: excessive correction may obscure biological variation, while insufficient correction leaves technical artifacts.

To address these persistent challenges, we present scMGCL (single-cell multi-omics graph contrastive learning), a framework that synergizes graph neural networks with contrastive learning for robust multi-omics integration. Our approach introduces three key innovations: (i) the construction of modality-specific cell similarity graphs that preserve both local and global cellular relationships; (ii) a contrastive learning framework that maximizes mutual information between matched cells across modalities while discriminating dissimilar cells; and (iii) efficient information delivery through graph convolutional networks (GCNs) that maintain intra-modality cellular relationships during integration.

Through a comprehensive evaluation on diverse datasets, we demonstrate that scMGCL achieves superior performance across multiple metrics. Specifically, scMGCL excels at: (i) accurately aligning cell populations across modalities; (ii) preserving biological consistency by constructing graph structures; and (iii) achieving computational efficiency with reduced runtime and memory usage for large datasets. Ablation studies confirm the importance of the graph-based architecture, while parameter sensitivity analyses offer guidelines for optimal performance. By addressing both the biological and computational challenges, scMGCL enables more accurate and scalable analysis of cellular states or types.

## 2 Materials and methods

### 2.1 scMGCL model

**Figure 1.**
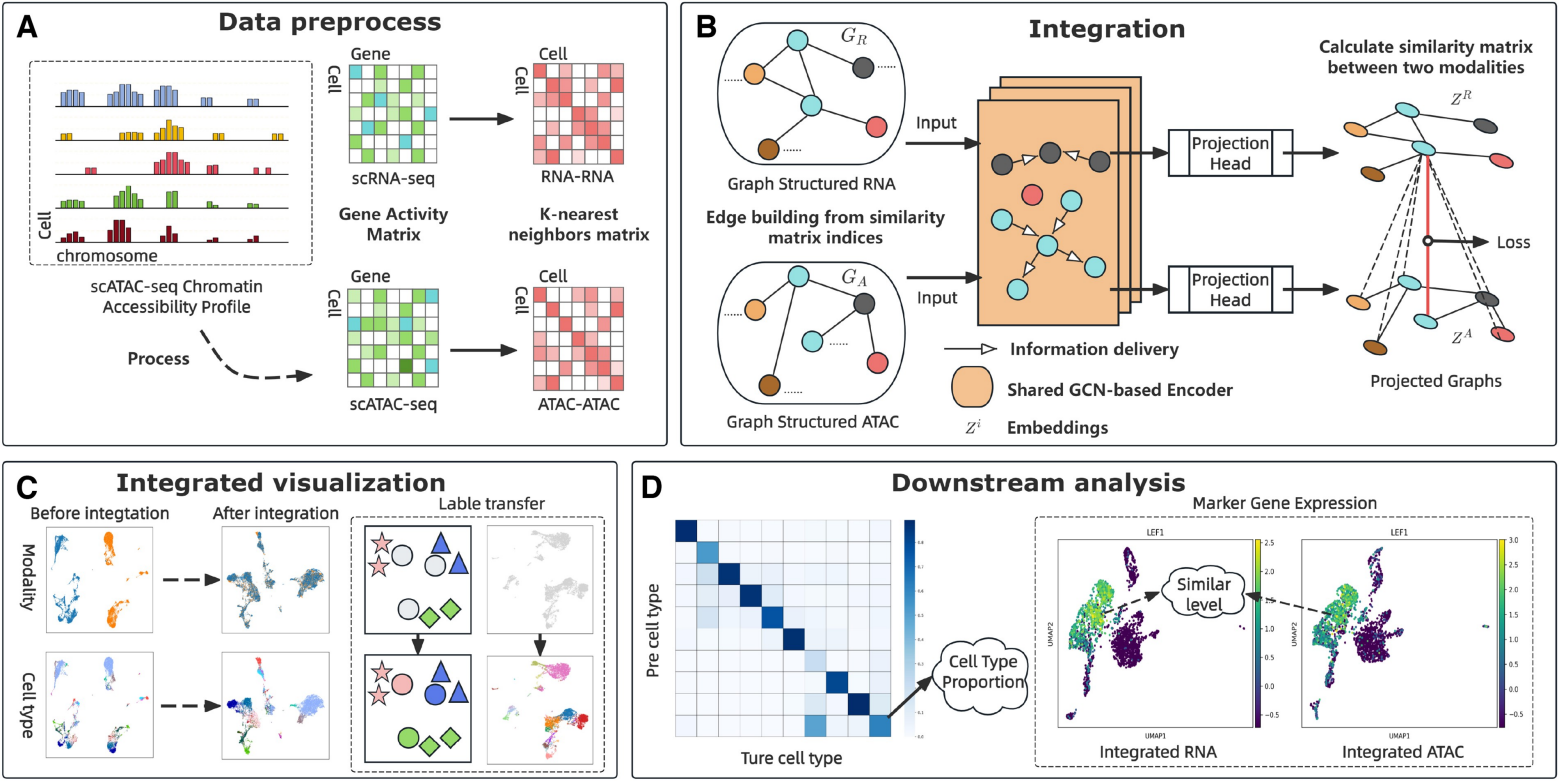
Overview of the scMGCL workflow. (A) Transform scATAC-seq peaks into a gene activity matrix to build adjacency relationships. Construct *k*-nearest neighbor (KNN) graphs separately for preprocessed RNA and ATAC data, resulting in modality-specific adjacency matrices. (B) Construct the input graph structure based on these relationships. The shared GCN-based encoder enhances cell clustering, while contrastive loss minimizes distances between similar cells. (C) Perform UMAP visualization on both unintegrated and integrated datasets to evaluate modality mixing and cluster preservation. Transfer cell type labels from RNA-to-ATAC cells using KNN classification on the integrated data (only ATAC cells are shown for clarity). (D) Verify label transfer accuracy by calculating transfer ratios on the integrated data. Additionally, assess integration effectiveness by comparing marker-gene expression levels across modalities.

To achieve rapid, stable, and high-performance integration of multi-omics matched data, we develop scMGCL, a framework that synergizes graph neural networks with cross-modality contrastive learning (Fig. 1). In single-cell data, gene expression profiles from cells of the same type tend to share similar patterns (Stuart *et al.* 2019). This observation aligns with the principle of contrastive learning, which encourages the model to maximize similarity between positive sample pairs (e.g. the same cell across modalities) and minimize similarity with negative pairs. By learning to emphasize shared patterns, contrastive learning promotes robust feature extraction while reducing sensitivity to noise or irrelevant variations.

The method operates through three key phases: (i) graph construction: for each modality (RNA-seq and ATAC-seq), scMGCL constructs *k*-nearest neighbor graphs based on cell similarity metrics, preserving both local and global cellular relationships; (ii) joint embedding: GCNs project these modality-specific graphs into a shared low-dimensional space, maintaining topological structure while learning latent representations; and (iii) cross-modality contrast: unlike traditional graph contrastive learning that applies artificial augmentations to single graph, scMGCL treats each modality’s intrinsic graph as a natural augmentation for the other. This strategy leverages biologically meaningful relationships rather than synthetic perturbations. In single-cell multi-omics, both RNA and ATAC-derived gene activity matrices encode cellular states as high-dimensional gene-feature vectors (Vandereyken *et al.* 2023). This structure is conceptually similar to sentences in natural language processing (NLP), where genes act as vocabulary elements and their expression patterns form cell-type-specific “phrases”. Cells of the same type typically exhibit recurring patterns of gene expression. Contrastive learning enables the model to capture both local relationships (within a modality) and global correspondences (across modalities) without relying on labeled data. Given that most single-cell multi-omics datasets lack comprehensive annotations, contrastive learning is particularly well suited for learning biologically meaningful representations in an unsupervised manner.

The framework’s dual optimization mechanism ensures complementary learning: GCNs enhance intra-modality clustering by propagating features across graph edges. At the same time, contrastive loss aligns inter-modality cell pairs through a noise-resistant objective function. This synergistic approach addresses two critical challenges in multi-omics integration—preserving modality-specific biological signals while aligning shared cellular states.

### 2.2 Graph data construction

To address the complex relationships between genes within modalities during the integration process, we simulate their structure by constructing a graph G from the data at the modal level. In this graph, cells are represented as vertices $V={\left\{v_{i}\right\}}_{i=1}^{N}$ and the *k*-nearest neighbors algorithm is used to identify the *k* closest cells to each cell vertex, connecting them with edges. In GNNs, these edges facilitate information transmission, causing different types of cells within a modality to cluster based on this propagation. This approach, combined with a visualization that provides greater discrimination, enables more distinct and interpretable clustering of cell types.

We transform the sparse, high-dimensional, and noisy scATAC-seq peak data into a biologically meaningful gene activation matrix (Stuart *et al.* 2021) after quality control, gene annotation, and normalization. This conversion better reflects gene regulatory dynamics. For both single-cell RNA and ATAC data, we perform essential preprocessing steps: normalization to ensure uniform expression, logarithmic transformation to stabilize variance and reduce the impact of highly expressed genes, identification of highly variable genes (HVGs) (Amezquita *et al.* 2020) to capture key biological information, and feature standardization. Dimensionality reduction is also applied to retain as much original information as possible, ensuring the data are optimally prepared for graph construction and analysis.

For the processed data, we calculate the distance matrix of cells from the same modality using Euclidean distance:


(1) $$d_{ij}=\sqrt{\Sigma_{k=1}^{d}{\left(x_{ik}-x_{jk}\right)}^{2}}$$

where $d_{ij}$ represents the Euclidean distance between cell *i* and cell *j*, $x_{ik}$ and $x_{jk}$ are the feature values of the *i*th and *j*th cells, respectively, and *d* is the number of features.

From this distance matrix, we retain the top *k* closest pairs of cells and use these as edge indices to construct the graph:


(2) $$\mathrm{Edge}\;\mathrm{Index}=\{(i,j)|d_{ij}\leq \mathrm{threshold}\}$$

where *threshold* is determined by the top *k* smallest distances.

At this stage, we have constructed the graph structures for the two omics data, which we denote as $G_{i}=\{V_{i},E_{i}\}$. When i=R, $G_{i}$ represents the graph structure of RNA, where $V_{i}$ and $E_{i}$ denote the set of nodes and edges of RNA. When i=A, the same notation applies to ATAC.

Due to the construction of edges based on a fixed number of the top *k* most similar cells, when there are fewer cells of the same type, cells from dissimilar cell types may inadvertently be included in edge construction. This can lead to suboptimal information delivery. Therefore, for the constructed edge indices, we randomly remove a portion of unidirectional edges to mitigate this effect.

### 2.3 Model architecture

We designed a graph embedding strategy to capture high-level feature representations while preserving structural information. This approach utilizes a graph convolutional projection encoder, consisting of two key components: an enhanced graph convolutional neural network encoder and a contrastive learning projector.

#### 2.3.1 GCN encoder

In this study, we use a three-layer GCN to encode single-cell multi-omics data. The first two layers of the GCN are followed by a ReLU activation function. The third layer does not have an activation function, as the subsequent projector includes one. This design choice results in a more compact visualization compared to using an activation function in the final layer.

The initial input to the GCN consists of the data matrix *X* and edge indices *E*. The GCN constructs the adjacency matrix $\hat{A}$ based on the node set and edge indices, which is then normalized as follows:


(3) $$\hat{A}=D^{-\frac{1}{2}}AD^{-\frac{1}{2}}$$

where *D* is the degree matrix with diagonal elements $D_{ii}$ representing the degree of node *i*.

- The first two layers of GCN could be formulated as:
  (4) $$H^{\left(l+1\right)}=\sigma (\hat{A}H^{\left(l\right)}W^{\left(l\right)})$$

where *l* denotes the layer number, with $H^{0}=X$, where *X* is the input feature matrix and $W^{\left(l\right)}$ is the weight matrix for layer *l*. The function σ represents the ReLU activation function.

- The third layer of GCN is as follows:
  (5) $$H^{\left(3\right)}=\hat{A}H^{\left(2\right)}W^{\left(2\right)}$$

The ReLU activation function is omitted to create a more compact representation. The feature matrix $H^{\left(3\right)}$ obtained from the three-layer GCN is then used in the subsequent contrastive learning framework to maximize the representation similarity of the same cell across different modalities.

#### 2.3.2 Contrastive learning projector

We designed a projector to project the representations output by the GCN encoder into a new representation space for contrastive learning. The projection head consists of two fully connected layers and a ReLU activation function:

- First fully connected layer: this layer maps the high-dimensional features *H*
 
  (6) $$h=\mathrm{ReLU}(W^{\left(p1\right)}H^{\left(3\right)})$$
- Second fully connected layer maps the hidden dimension features *h* to the final output dimension:
  (7) $$z=W^{\left(p2\right)}h$$

where *z* represents the final output representations.

### 2.4 Model training

We utilize the RNA and ATAC views as training samples, treating cells in the same position as positive sample pairs and cells in different positions as negative sample pairs. During training, we compute the normalized similarity matrix and focus on increasing the values at diagonal positions, thereby maximizing the similarity of positive sample pairs. This approach implicitly reduces the similarity between negative sample pairs, facilitating the integration of corresponding cells across modalities. The contrastive loss function is defined as follows:


(8) $$L=-\frac{1}{N}\Sigma_{i=1}^{N}\;\text{log}\;\frac{\;\text{exp}(\mathrm{sim}(z_{i}^{R},z_{i}^{A})/\tau )}{\Sigma_{j=1}^{N}\;\text{exp}(\mathrm{sim}(z_{i}^{R},z_{j}^{A})/\tau )}$$

where $z_{i}^{R}$ and $z_{i}^{A}$ are the representations of cell *i* in the RNA and ATAC views, respectively, sim(·,·) denotes the cosine similarity, and τ is a temperature parameter.

The graph-structured data $G_{A}$ (ATAC) and $G_{R}$ (RNA) are input into a pre-trained model consisting of three-layer GCNs followed by a projection head. This produces low-dimensional embeddings $Z_{A}$ and $Z_{R}$, which are then concatenated by cell identifiers to form a unified embedding *Z*, integrating features from both modalities. To analyze the combined embedding, we construct a *k*-nearest neighbor graph using *scanpy*.*pp*.*neighbors* (Wolf *et al.* 2018) with n_neighbors = 30, followed by UMAP for non-linear dimensionality reduction using *scanpy*.*tl*.*umap*. The resulting visualization is colored by modality (RNA/ATAC) and cell type to illustrate the alignment of multi-omic features and preservation of biological states.

## 3 Results

To evaluate the performance of scMGCL, we conducted experiments on diverse datasets spanning multiple species and varying sizes, comparing it with state-of-the-art baseline methods. The results demonstrate the robustness and superiority of the scMGCL integration performance.

*Datasets*: To evaluate the effectiveness of our proposed method, we collected four paired datasets measuring both DNA accessibility and gene expression data: (i) the SNARE-seq (Chen *et al.* 2019) dataset from mouse brain, and (ii) three human PBMC datasets—PBMC3k (Satija *et al.* 2015), PBMC10k (Hao *et al.* 2021), and PBMCMultiome (Satija *et al.* 2015). All datasets across methods were uniformly preprocessed using the Signac (Stuart *et al.* 2021) pipeline. For detailed descriptions of the datasets and specific preprocessing procedures, please refer to the Supplementary Notes (Data preprocess), available as supplementary data at *Bioinformatics* online.

*Baselines*: We conducted a comprehensive comparison with seven state-of-the-art integration tools across multiple paired RNA–ATAC datasets: MultiMAP (Jain *et al.* 2021) employs graph-based manifold alignment for modality fusion, SCALEX (Xiong *et al.* 2022) uses variational autoencoders with latent space alignment, Scanorama (Hie *et al.* 2019) corrects batches via mutual nearest neighbors, scCorrector (Guo *et al.* 2024) performs nonlinear matrix decomposition, uniPort (Cao *et al.* 2022) implements adversarial training with hybrid regularization, Harmony (Korsunsky *et al.* 2019) applies PCA-based linear correction, and Seurat (Hao *et al.* 2021) relies on CCA and anchor-based integration, across multiple paired RNA-ATAC datasets. The principle of baselines and the parameter settings can be found in the Supplementary Notes (Baseline methods), available as supplementary data at *Bioinformatics* online.

*Integration performance metrics*: We integrated scRNA-seq and scATAC-seq data, training a classifier to predict ATAC cell types. We evaluated the model using the following metrics: adjusted rand index (ARI) for clustering similarity, normalized mutual information (NMI) for alignment with true labels, and *F*1 scores (*F*1) for a balanced measure of precision and recall. The sum of these metrics (SUM) was used for overall comparison and effectiveness assessment. The Batch Entropy (BE) score is used to evaluate the extent of mixing cells across datasets, and the Silhouette coefficient (SI) is used to evaluate the separation of biological distinctions. For definitions and equations of all metrics, please see the Supplementary Notes (Integration performance metrics), available as supplementary data at *Bioinformatics* online.

### 3.1 scMGCL efficiently integrates paired datasets while maintaining stability

**Figure 2.**
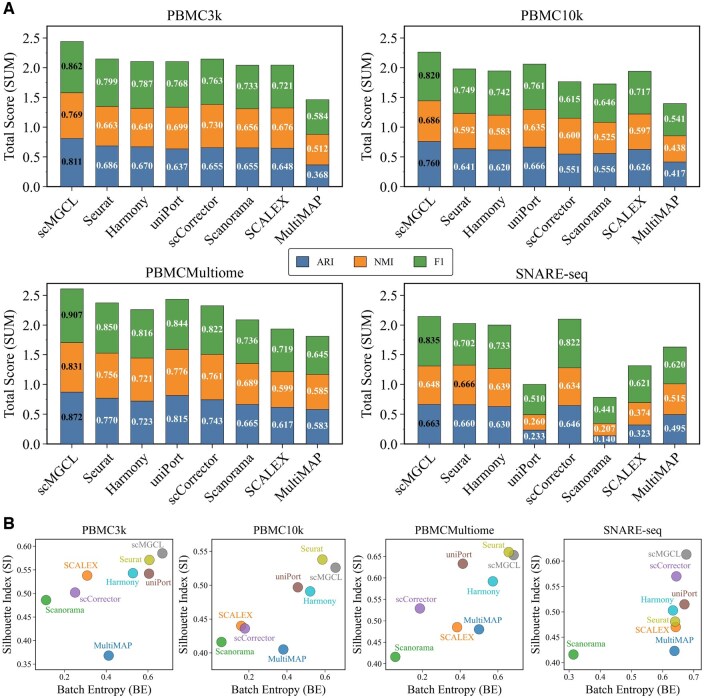
Performance of scMGCL and benchmark methods on single-cell multi-omics datasets. (A) Performance comparison of each method across datasets, evaluated using *ARI*, *NMI*, and *F*1 score. The summation of these metrics is represented as the *SUM* score, with the highest value for each metric highlighted in black to emphasize comparative superiority. (B) Comparison of Batch Entropy (*BE*) and Silhouette Index (*SI*) for each method across all datasets.

We evaluated scMGCL’s performance on paired single-cell RNA-seq and ATAC-seq datasets spanning human peripheral blood mononuclear cells (PBMC3k, PBMC10k, and PBMCMultiome), and mouse brain cells (SNARE-seq). Benchmarking against state-of-the-art methods revealed scMGCL’s consistent superiority: it achieved the highest scores in most evaluation metrics (*ARI*, *F*1, *SUM*, and *BE*) across all datasets (Fig. 2). In the remaining cases, scMGCL ranked second with marginal differences (0.01–0.02), demonstrating robust stability compared to method-specific performance fluctuations observed in other tools.

Notably, both uniPort and SCALEX showed comparable and stable *SUM* scores on PBMC datasets but failed on the SNARE-seq dataset (△SUM> 0.82 versus scMGCL), indicating limited cross-species applicability (Fig. 2A). Similarly, scCorrector exhibited instability on PBMC10k (△SUM> 0.47 versus scMGCL). MultiMAP exhibited suboptimal integration (SUM< 1.82) on all datasets, while Scanorama showed significant dataset-dependent variation, particularly underperforming on SNARE-seq (△SUM= 1.26 versus PBMC3k). Although Harmony and Seurat demonstrate moderate performance (△SUM< 0.35 versus scMGCL), Harmony struggled with fine-grained subtype resolution (Fig. S1, available as supplementary data at *Bioinformatics* online), and Seurat incompletely aligned PBMC10k cell populations (Fig. S2, available as supplementary data at *Bioinformatics* online). Benchmark methods generally showed performance declines on the SNARE-seq dataset due to inherent differences between mouse and human cellular features. In contrast, scMGCL consistently demonstrated superior and scalable performance across species.

scMGCL’s biological fidelity was demonstrated through two key metrics (Fig. 2B): (i) Batch Entropy (*BE*): scMGCL consistently achieved perfect scores across all datasets, indicating robust modality mixing without over-correction. In contrast, Scanorama exhibited particularly poor modality mixing (△BE> 0.362 versus scMGCL). MultiMAP, SCALEX, and scCorrector showed significant dataset-dependent fluctuations, ranging from scCorrector’s best performance (0.644 on SNARE-seq) to SCALEX’s lowest (0.160 on PBMC10k). While Harmony and Seurat demonstrated stable cross-modality integration (BE> 0.5), scMGCL consistently outperformed all competing methods while maintaining high stability (BE> 0.65); (ii) Silhouette Coefficient (*SI*): scMGCL ranked first in PBMC3k (0.585) and SNARE-seq (0.613), with near-optimal performance on PBMC10k (0.526 versus Seurat’s 0.538) and PBMCMultiome (0.653 versus Seurat’s 0.660). While Seurat performed comparably to scMGCL on PBMC datasets, it significantly underperformed on SNARE-seq data (△SI= 0.132 versus scMGCL). MultiMAP and Scanorama showed suboptimal cell-type discrimination (0.368–0.486), while SCALEX and scCorrector exhibited instability, particularly on PBMC10k. uniPort and Harmony maintained moderate consistency (SI> 0.49), but scMGCL achieved superior cell-type discrimination (0.526–0.653) across diverse datasets and species, demonstrating its robust and scalable performance.

**Figure 3.**
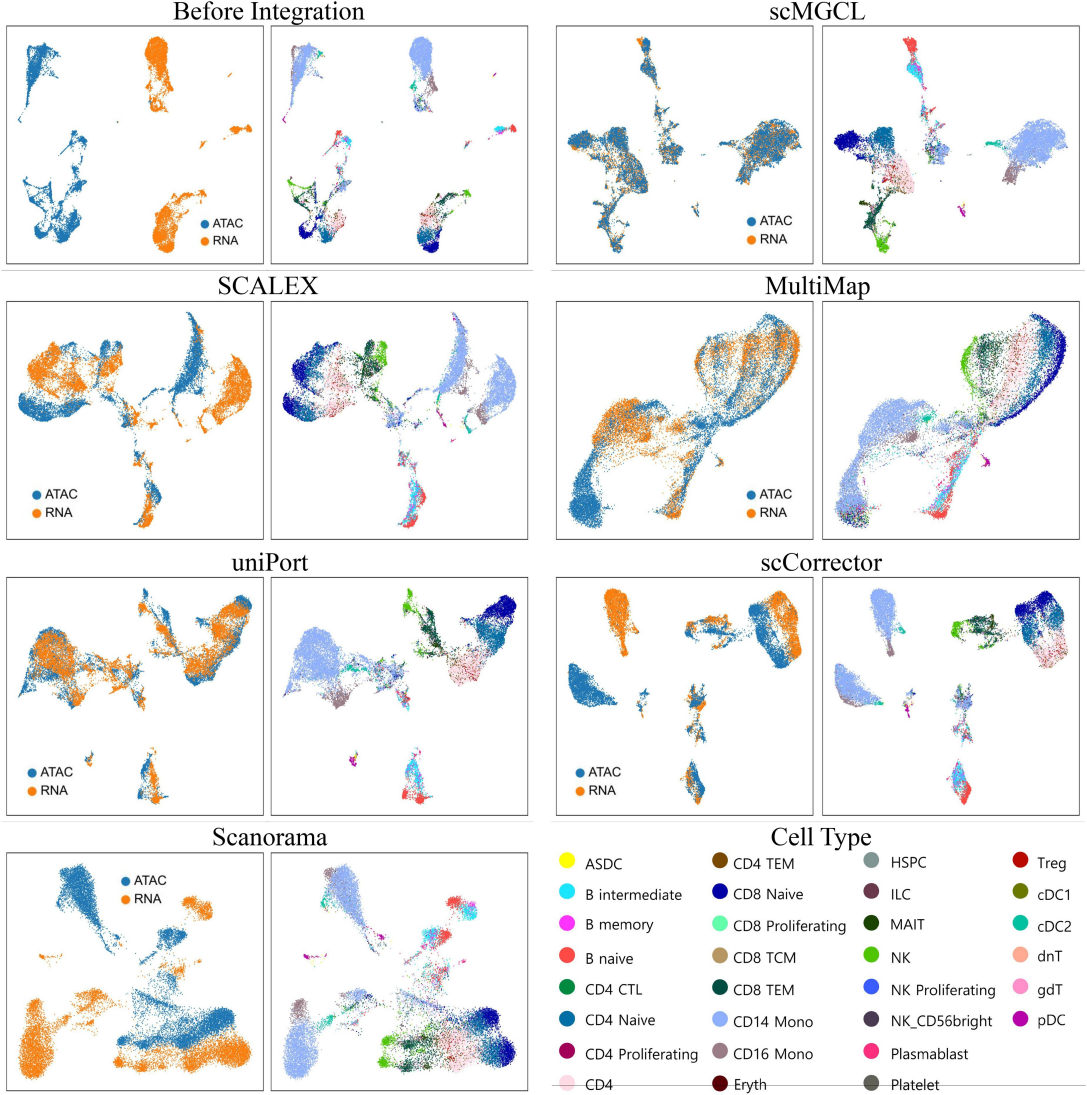
UMAP visualization comparing integration results of scMGCL and benchmark methods on PBMC10k dataset. The left side of each subgraph displays the clustered results for the two single omics (RNA and ATAC). On the right side, the cell types are clustered and colored according to the original labels (top left).

Visual inspection of UMAP embeddings supported these findings. scMGCL consistently demonstrated near-perfect overlap between RNA and ATAC modalities, forming distinct, homogeneous cell-type clusters across all datasets [PBMC10k (Fig. 3), PBMC3k (Fig. S3, available as supplementary data at *Bioinformatics* online), PBMCMultiome (Fig. S4, available as supplementary data at *Bioinformatics* online), and SNARE-seq (Fig. S5, available as supplementary data at *Bioinformatics* online)]. In contrast, competing methods exhibited either poor modality alignment (e.g. Scanorama and scCorrector, with low BE scores) or indistinct cluster separation (e.g. Scanorama and MultiMAP, with reduced SI values, Fig. 2A). Notably, SCALEX and scCorrector show a mismatch between quantitative metrics and visualization quality. Despite showing competitive label transfer accuracy (Figs 2A and 3), their UMAP plots revealed persistent modality gaps (BE< 0.2; Fig. 2B), emphasizing the necessity for multimodal validation.

**Figure 4.**
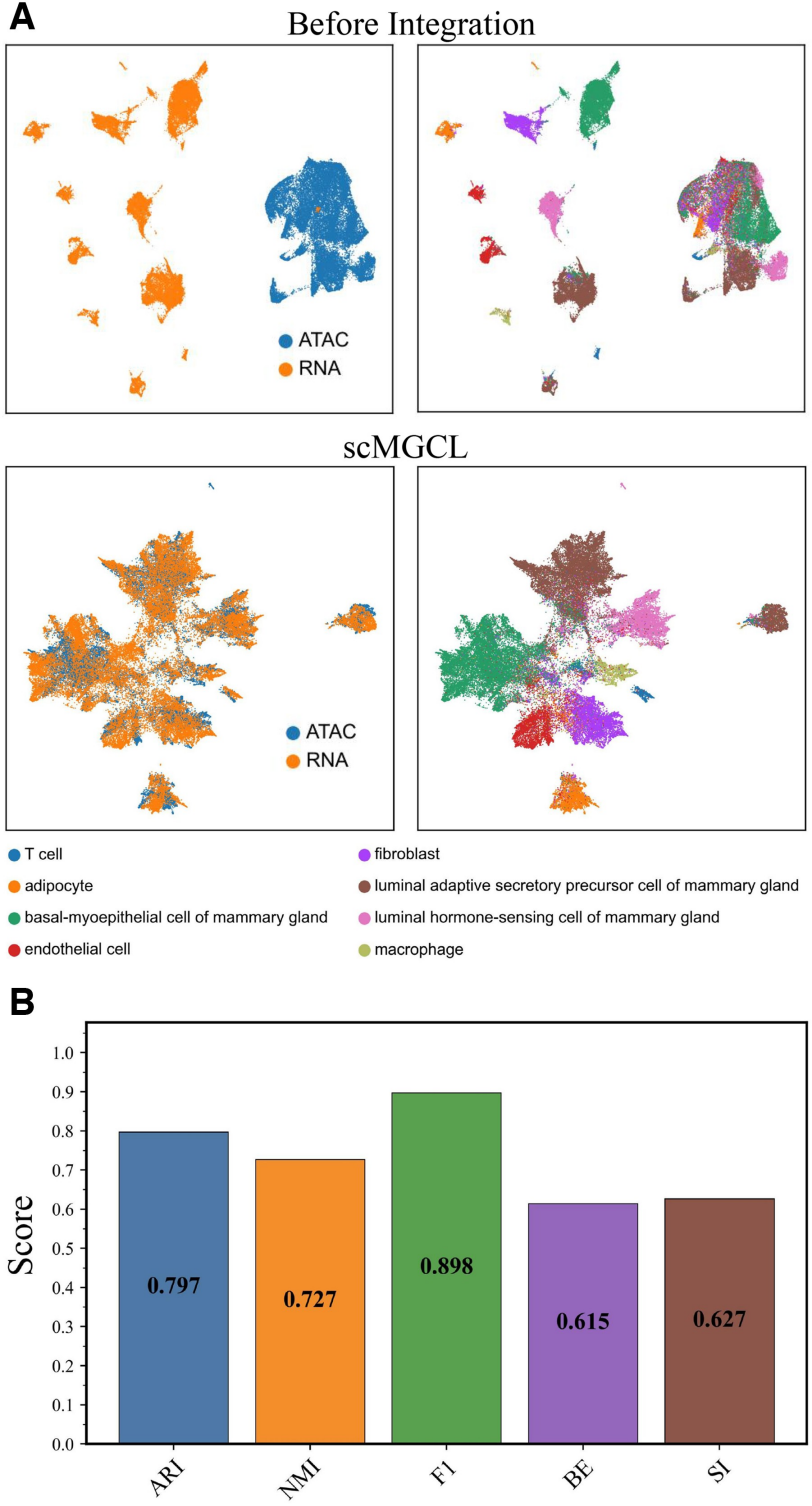
Integration performance of a scMGCL on single-nucleus multi-omics dataset. (A) UMAP visualization comparing pre-integration and scMGCL-integrated results. In each subpanel, the left plots show clustering by modality (RNA and ATAC), while the right plots display integrated clustering colored by original cell types (bottom). (B) Quantitative performance metrics of scMGCL on the single-nucleus multi-omics datasets.

We analyzed 51 367 paired single-nucleus RNA sequencing (snRNA-seq) and single-nucleus ATAC sequencing (snATAC-seq) profiles derived from breast tissues of healthy women with diverse genetic ancestry, generated using the 10× Multiome platform. The integrated results are shown in Fig. 4.

The UMAP visualization (Fig. 4A) shows that scMGCL effectively integrates the two modalities, achieving accurate alignment and clear clustering that reflects underlying cellular heterogeneity. This is supported by *BE* and *SI* both exceeding 0.6 (Fig. 4B). The *ARI* of 0.797 further confirms the success of label transfer, highlighting the robustness of the integration. Additionally, scMGCL demonstrates scalability across different cell numbers, species, and both nuclear and cytoplasmic transcriptomes.

### 3.2 scMGCL enables accurate RNA-to-ATAC label transfer

**Figure 5.**
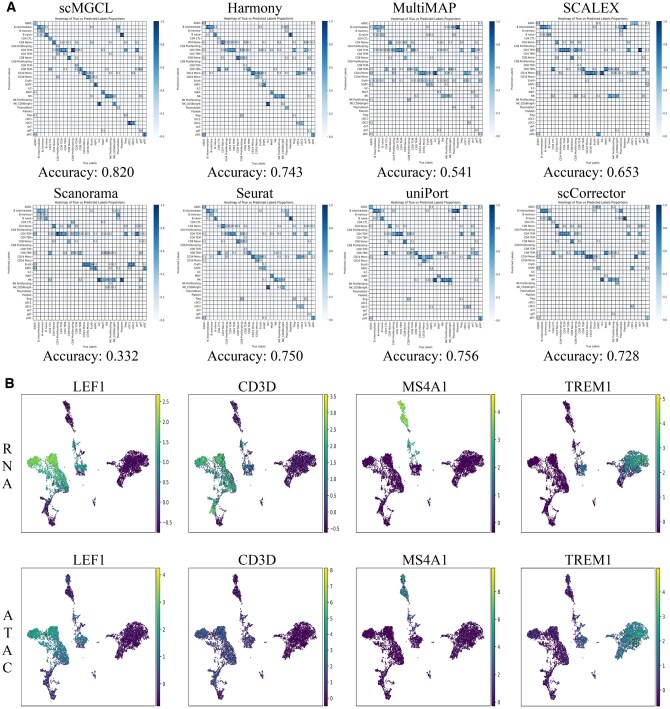
Analysis of the integrated dataset in label transfer and integration validation. (A) Confusion matrix heatmap of true and predicted cell types for the PBMC10k dataset. The matrix shows true cell types on the *x*-axis and predicted cell types on the *y*-axis. The color intensity of each tile represents the proportion of cells from a true type classified as each predicted type. (B) Marker-gene expression (LEF1, CD3D, MS4A1, TREM1) in the integrated ATAC cells and RNA cells for the PBMC10k dataset.

To evaluate cross-modality biological consistency, we performed RNA-to-ATAC cell-type label transfer using KNN classification on integrated embeddings. scMGCL outperformed baseline methods, achieving higher accuracy in predicting various cell types on PBMC10k (Fig. 5A). Detailed metrics for the three additional datasets are provided in Figs S6 (PBMC3k), S7 (PBMCMultiome), and S8 (SNARE-seq), available as supplementary data at *Bioinformatics* online. While benchmarking methods showed variable performance—Scanorama, SCALEX, and MultiMAP performed well only for abundant cell types (e.g. CD14+ monocytes)—scMGCL maintained >70% accuracy even for rare populations (<2% prevalence, e.g. dendritic cells) in PBMC datasets. Additionally, it achieved over 80% accuracy for rare cell types (<3% prevalence, e.g. oligodendrocytes) in the SNARE-seq dataset. Methods with moderate overall performance (Harmony, Seurat, and uniPort) exhibited subtype-specific failures: Harmony and Seurat misclassified 50%–60% of CD16+ monocytes as CD14+ monocytes, while uniPort incorrectly labeled 80% of CD56bright NK cells as conventional NK cells (Fig. 5A). Accuracy for plasmacytoid dendritic cells (pDC) varied widely across methods (0%–80% in PBMC3k and PBMC10k), but scMGCL consistently achieved high accuracy (>90%) in all PBMC datasets (PBMC3k, PBMC10k, and PBMCMultiome). Across all evaluated datasets, scMGCL demonstrated the highest Mean accuracy (>0.8), with peak performance on the PBMC Multiome dataset (0.902), outperforming the next-best methods, Seurat and uniPort, by a significant margin (△accuracy>5% versus scMGCL), demonstrating its robustness in cross-modal cell type annotation.

scMGCL’s robustness arises from its dual optimization strategy: (i) cross-modality contrastive learning treats RNA and ATAC as mutual augmentations, aligning shared biological signals while filtering technical noise; (ii) GCNs preserve multi-scale cellular relationships, maintaining local neighborhood structures essential for KNN classification. Edge-based information delivery further enhances cellular heterogeneity representation, improving cell subtype identification.

Biological validation through marker-gene analysis corroborated scMGCL’s effectiveness. In integrated ATAC embeddings, canonical markers accurately co-localized with their predicted cell types (Fig. 5B): (i) T cell maturation: *LEF*1 peaks concentrated in naive CD4/CD8 T cells, with significantly higher mRNA expression in naive T cells compared to memory T cells, and a positive correlation between expression levels and ATAC signal intensity (PBMC10k; Fig. 3); (ii) T cell signaling: *CD*3*D* was ubiquitously accessible across all T cell clusters, with stable mRNA expression across both developmental and functional T cells (PBMC3k; Fig. S9, available as supplementary data at *Bioinformatics* online); and (iii) Neuroglial function: *AQP*4 (astrocytes) and *SLC*17*A*7 (excitatory neurons) showed matching accessibility and RNA expression patterns in SNARE-seq (Figs S5 and S11, available as supplementary data at *Bioinformatics* online). A detailed explanation is provided in the Supplementary Notes (line 96), available as supplementary data at *Bioinformatics* online. Critically, marker-gene correlations between RNA expression and ATAC accessibility remained high following integration (*r *= 0.9, △r< 0.04 compared to original data; Table S2, available as supplementary data at *Bioinformatics* online), indicating that scMGCL effectively preserves multi-omics biological relationships. This consistency highlights the synergy between contrastive modality alignment and GCN-based feature propagation in achieving biologically faithful integration.

To quantify the extent of biological signal preservation, we conducted a comparative analysis across all methods. Specifically, we calculated the Pearson correlation between marker-gene accessibility profiles in the original and integrated datasets (Li *et al.* 2022). Biological signal loss was defined as (1 − correlation coefficient), reflecting the proportion of information degraded during integration.

As shown in Fig. S12, available as supplementary data at *Bioinformatics* online, scMGCL consistently demonstrated minimal biological signal loss across all datasets (l< 0.0497). In contrast, although some alternative methods performed well on specific genes, they often exhibited substantially higher loss (l> 0.1) on other datasets. Importantly, since label transfer relies on the preservation of gene expression patterns, inadequate retention of such features directly impaired cell type classification. For example, in PBMC3k dataset, methods exhibiting substantial signal loss for *LYZ* and *TREM*1 (notably Scanorama, scCorrector, and uniPort) misclassified CD16+ monocytes as CD14+ monocytes (Fig. S6, available as supplementary data at *Bioinformatics* online); in the PBMCMultiome dataset, scCorrector, which showed the highest signal loss for *TREM*1, misclassified over 90% of macrophages as conventional dendritic cells (Fig. S7, available as supplementary data at *Bioinformatics* online); in the SNARE-seq dataset, Scanorama—with 99.05% signal loss at the *Trf* locus—correctly identified only 10% of oligodendrocytes (Fig. S8, available as supplementary data at *Bioinformatics* online). These findings demonstrate that scMGCL most effectively preserves critical biological signals, resulting in superior label transfer accuracy and more faithful representation of cellular identity. The ability to retain marker-gene features directly enhances downstream biological interpretation, underscoring the importance of signal preservation in single-cell multi-omics integration.

### 3.3 scMGCL offers a fast and lightweight solution for paired multi-omics integration

We systematically assessed the computational efficiency of all methods using a standardized hardware setup. CPU-based methods were run on a 16 vCPU Intel (R) Xeon (R) Gold 6430 (32 cores, 120 GB DDR4 RAM), while GPU-based methods used an NVIDIA RTX 4090 (24 GB VRAM). All methods were evaluated on identical computational resource configurations, as detailed in Table S3, available as supplementary data at *Bioinformatics* online.

**Figure 6.**
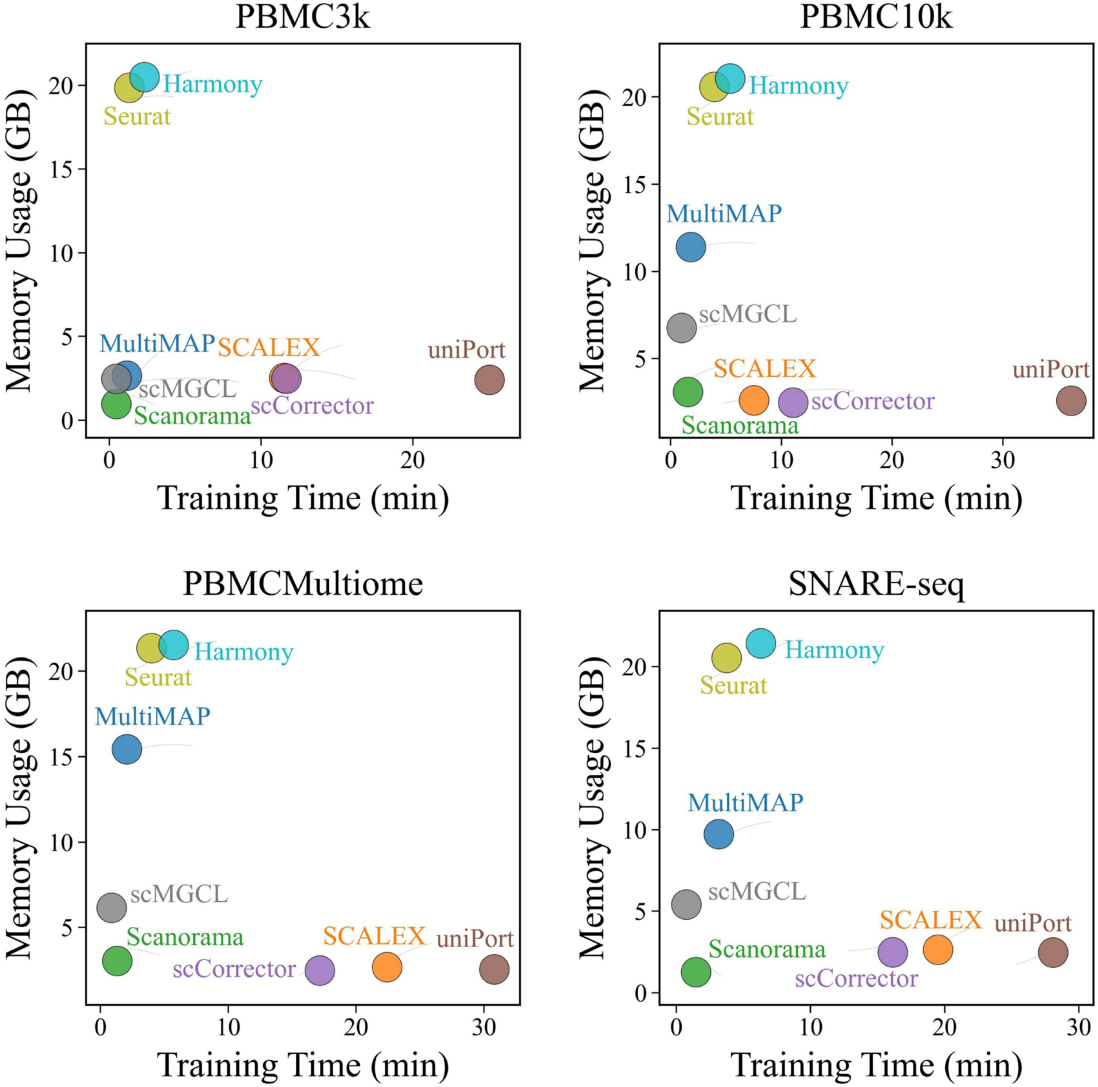
Comparative time consumption and memory usage of integration methods across datasets.

scMGCL demonstrated significant improvements in both runtime and memory efficiency compared to state-of-the-art tools (Fig. 6). On the PBMC3k dataset, scMGCL completed integration in 28.8 s (0.48 min) using 2.46 GB of memory—5× faster than CPU-based methods like Harmony (2.33 min) and 8× more memory-efficient than Seurat (19.84 GB). In contrast, GPU-accelerated tools like uniPort take over 25 min for equivalent analyses. scMGCL achieved over >98% reduction in computational time (mean: 0.78 min). While Scanorama was comparable in speed (29 s) and low memory usage (0.938 GB), its biological accuracy was lower (△ARI = 0.157 versus scMGCL). This performance-efficiency balance stems from scMGCL’s non-iterative contrastive learning framework and sparse graph optimizations, avoiding expensive adversarial training cycles while preserving biological fidelity.

### 3.4 Ablation studies

**Figure 7.**
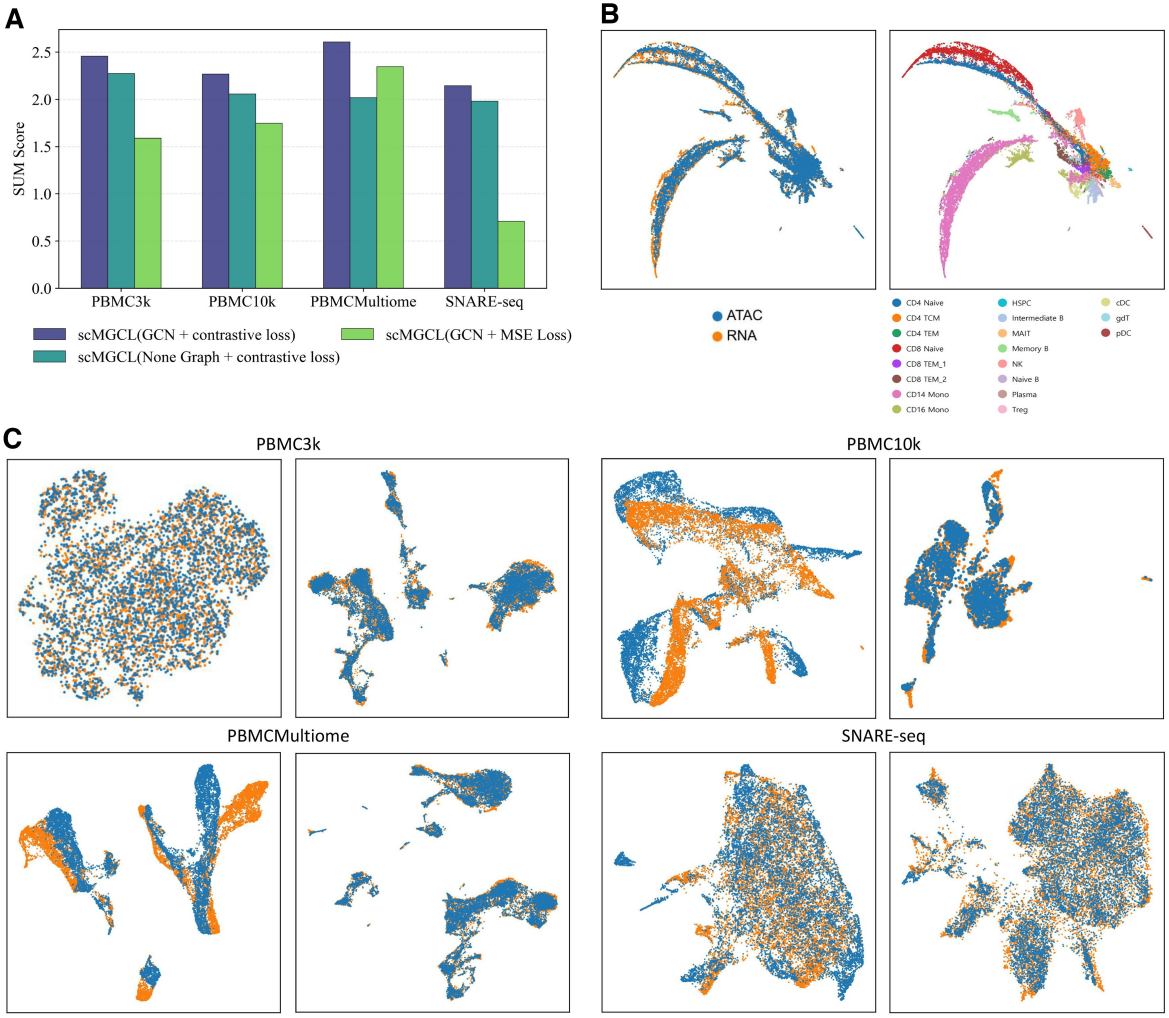
Integration performance of different ablated model versions across multiple datasets. (A) The SUM score for various ablated model versions is presented across multiple datasets, demonstrating the performance of each version. (B) UMAP visualization of PBMCMultiome dataset integrated by scMGCL(GCN + MSE loss), where cell types are color-coded based on annotations. (C) Visualization comparing non-graph and graph structures across datasets. On the left, scMGCL (None Graph + contrastive loss) integration results are shown, while on the right, scMGCL (GCN + contrastive loss) integration is displayed, highlighting the impact of graph structures.

To dissect scMGCL’s architectural contributions, we conducted systematic ablations evaluating two core components: graph structure encoding and contrastive loss formulation (Fig. 7).

#### 3.4.1 Graph structure

We replaced the GCN with a multilayer perceptron (MLP), keeping the overall model architecture consistent (three-layer), along with identical parameter settings, activation functions, projection head, and training protocol (Adam optimizer with contrastive loss). This modification removed explicit modeling of cell–cell relationships, resulting in a notable performance decline (△SUM> 0.165 compared to scMGCL (GCN + contrastive loss); Fig. 7A). Visualization of the MLP-based variant (scMGCL-MLP) revealed diffuse cluster boundaries (Fig. 7C), confirming that graph propagation enhances cellular discrimination by leveraging neighborhood relationships. This demonstrates that explicit topological modeling via GCNs is essential for preserving biological fidelity.

#### 3.4.2 Loss function

We further explored the impact of the loss function by replacing the contrastive loss with mean squared error (MSE) loss. Although both losses aim to minimize distances between positive cell pairs, this substitution alters the optimization landscape. Cross-entropy, a key component in contrastive loss, was replaced by MSE while all other setting were kept unchanged. The modified loss function is defined as:


(9) $$L_{\text{MSE}}=\frac{1}{N}\sum_{i=1}^{N}{\left(\frac{\;\text{exp}(\text{sim}(z_{i}^{R},z_{i}^{A})/\tau )}{\sum_{j=1}^{N}\;\text{exp}\;(\text{sim}(z_{i}^{R},z_{j}^{A})/\tau )}-1\right)}^{2}$$

where $z_{i}^{R}$ and $z_{i}^{A}$ are the representations of cell *i* in the RNA and ATAC views, respectively, sim(·,·) denotes the cosine similarity, and τ is a temperature parameter.

Substituting the contrastive loss with MSE yielded biologically implausible integrations (scMGCL(GCN + MSE loss)) (e.g. 0.709 on SNARE-seq, Fig. 7A). While quantitative metrics suggested modest improvements on PBMCMultiome dataset, qualitative inspection exposed critical failures: over-smoothed embeddings obscured cell subtypes (Fig. 7B). This highlights the contrastive loss’s dual role in preventing overfitting while maintaining discriminative power.

We also evaluated the impact of different nearest neighbor construction methods (Fig. S18, available as supplementary data at *Bioinformatics* online) and different similarity matrix calculation methods (Fig. S19, available as supplementary data at *Bioinformatics* online) on the integrated performance, as well as the integrated performance of different enhancement methods in the contrastive learning step (Figs S20 and S21, available as supplementary data at *Bioinformatics* online). We employed an optimized computational pipeline utilizing KNN for efficient clustering, Euclidean distance metrics for constructing similarity matrices that consistently enable high-performance downstream integration, and cross-modality enhancement as our contrastive learning strategy, achieving robust integration performance while maintaining computational efficiency. For detailed analysis, please refer to the Supplementary Notes (line 110), available as supplementary data at *Bioinformatics* online.

These experiments conclusively validate scMGCL’s design: graph-based feature propagation and contrastive modality alignment synergistically enable robust, interpretable integration.

### 3.5 Parameter sensitivity analysis

**Figure 8.**
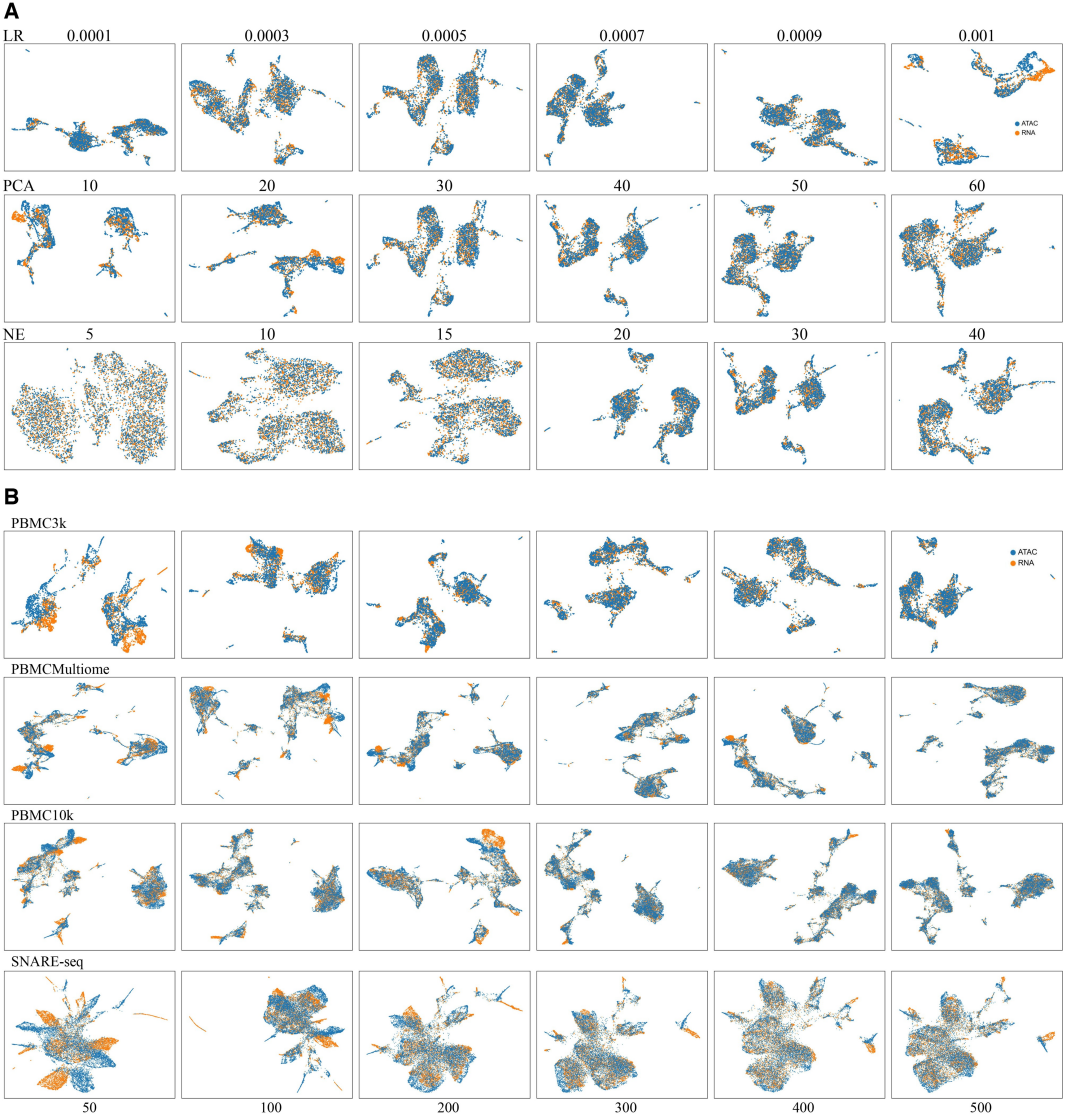
Performance of scMGCL with varying parameters across datasets. (A) UMAP visualization of scMGCL integration results on PBMC3k dataset under varying learning rates (LRs), PCA dimensions, and numbers of neighboring edges (NEs). This subfigure illustrates how changes in these parameters affect the quality of integration and clustering. (B) scMGCL integration performance with varying hidden dimensions (ranging from 50 to 500) across all datasets. This subfigure demonstrates how the number of hidden dimensions influences the overall integration performance, providing insight into the optimal architecture for scMGCL.

We systematically evaluated four hyperparameters [learning rate (LR), PCA dimensionality (PCA), number of neighboring edges (NE), hidden layer size, and temperature parameter (τ)] to optimize scMGCL’s performance. Quantitative *SUM* trends for LR and PCA across all datasets are shown in Figs S13A (LR) and S13B (PCA) , available as supplementary data at *Bioinformatics* online. Integration quality for varying LR, PCA, and NE was assessed via UMAP visualizations for all datasets [PBMC3k (Fig. 8A), PBMC10k (Fig. S14, available as supplementary data at *Bioinformatics* online), PBMCMultiome (Fig. S15, available as supplementary data at *Bioinformatics* online), and SNARE-seq (Fig. S16, available as supplementary data at *Bioinformatics* online)]. Hidden layer dimensions effects across datasets were compared via UMAP embeddings in Fig. 8B, while temperature parameter tuning dynamics are visualized in Fig. S17, available as supplementary data at *Bioinformatics* online.

Learning rate (LR): Lower values (LR < 0.0005) resulted in incomplete integration in PBMC10k (Fig. S14, available as supplementary data at *Bioinformatics* online) and suboptimal *SUM* across datasets (Fig. S13A, available as supplementary data at *Bioinformatics* online), while higher values (LR > 0.0007) caused over-smoothing (e.g. PBMC3k, Fig. 8A). We identified LR = 0.0006 as the optimal balance, achieving superior integration performance and robustness.

PCA dimensionality reduction (PCA): Integration was incomplete with PCA dimensionalities below 20, with performance stabilizing between 20 and 30 dimensions (e.g. PBMC3k, Fig. 8A). While PBMC datasets showed stable performance beyond 20 dimensions (△SUM< 0.05 versus 20 PCA; Fig. S13B, available as supplementary data at *Bioinformatics* online), SNARE-seq exhibited a performance decline beyond 30 dimensions (△SUM> 0.33 versus 20 PCA). We selected a unified PCA dimensionality of 30 to preserve cross-dataset biological variation while minimizing technical noise.

Number of Neighboring Edges (NE): Sparse graphs (NE < 15) resulted in segregated clusters (Fig. 8A and Figs S14 and S15, available as supplementary data at *Bioinformatics* online), while dense graphs (NE > 20) caused overfitting in PBMC10k (Fig. S15, available as supplementary data at *Bioinformatics* online) and SNARE-seq (Fig. S16, available as supplementary data at *Bioinformatics* online). NE = 20 provided the optimal balance, maximizing cross-modality connectivity without sacrificing resolution.

Hidden dimension: We explore this effect by gradually increasing the hidden layer dimension across datasets. Below 300 dimensions, all datasets failed to adequately capture multimodal features, resulting in fragmented UMAP embeddings (Fig. 8B). Beyond 300 dimensions, integration quality stabilized with negligible performance gains. We selected 300 dimensions to balance biological fidelity and computational efficiency.

Temperature parameter (τ): A temperature value that is too low (τ< 0.1) leads to incomplete modality alignment (e.g. PBMC10k, Fig. S17, available as supplementary data at *Bioinformatics* online), where the model struggles to reconcile heterogeneous features across modalities. In contrast, an excessively high temperature coefficient (τ> 0.2) results in oversimplified clustering and potential overfitting (e.g. PBMC3k, Fig. S17, available as supplementary data at *Bioinformatics* online), as the loss function over-smooths important discriminative features. Based on these observations, we selected τ= 0.1 as the optimal configuration.

## 4 Discussion

Integrating multimodal single-cell datasets offers both a transformative opportunity and a formidable computational challenge in modern biology. Although existing tools have laid the foundation for joint analysis of paired omics data, they often struggle with two persistent limitations: (i) loss of subtle but biologically critical cell states during integration, and (ii) the inability to maintain consistent performance across diverse tissue types and species. scMGCL addresses these challenges by combining graph neural networks and self-supervised learning, achieving both high accuracy and computational efficiency.

Our framework introduces several key advances over current methods. First, by reformulating modality integration as a graph contrastive learning problem, scMGCL overcomes the limitations of earlier dimensionality reduction and integration approaches (e.g. Seurat, Harmony). The graph architecture preserves neighborhood relationships essential for identifying cell types, as demonstrated by its higher label transfer accuracy. Second, the cross-modality augmentation strategy offers a biologically grounded alternative to artificial data perturbation techniques used in single-modality contrastive learning. This is particularly valuable for ATAC–RNA integration, where technical noise profiles differ between modalities (Zhang *et al.* 2022).

Recent advances in foundation models—such as scGPT (Cui *et al.* 2024), GeneFormer (Theodoris *et al.* 2023)—have shown remarkable performance in single-cell analysis. These pre-trained models produce informative low-dimensional embeddings that better capture cellular characteristics, thereby improving integration outcomes. In our study, we applied scGPT to generate embeddings for the PBMCMultiome dataset and concatenated them with the original features as input to scMGCL for multi-omics integration (Fig. S22, available as supplementary data at *Bioinformatics* online). Compared to the original unintegrated data (Before Integration; Fig. S4, available as supplementary data at *Bioinformatics* online), the scGPT-derived embeddings more effectively represent joint characteristics of the RNA and ATAC modalities (Fig. S22A, available as supplementary data at *Bioinformatics* online), as reflected by consistent topological structures and aligned cell-type distributions. This improved alignment supports more reliable identification of cross-modal shared features (Liu *et al.* 2021). Furthermore, when compared to scMGCL integration without scGPT augmentation (scMGCL; Fig. S4, available as supplementary data at *Bioinformatics* online), the combined approach achieves tighter clustering (Fig. S22B, available as supplementary data at *Bioinformatics* online), smoother cluster boundaries (△SI=0.08 versus scMGCL), and consistent performance gains metrics (△score>0.08). These results suggest that integrating foundation models with downstream methods such as scMGCL is a promising strategy for enhancing multi-omics single-cell data integration.

While scMGCL performs well with paired data, extending it to unpaired multi-omics integration requires further development, especially in managing modality-specific batch effects without matched cells. Additionally, adapting to emerging modalities (e.g. methylation, protein abundance) will require architectural changes to handle their unique noise profiles and feature spaces. We envision three strategic improvements: generalized alignment strategies for partially matched or unmatched datasets, dynamic graph construction to adjust inter-modality relationships, and multi-task learning to integrate over two modalities simultaneously.

As single-cell technologies evolve toward more complex multi-omic profiling, tools like scMGCL that can integrate complementary data layers will be essential for unlocking their full biological potential. Our approach not only provides immediate practical benefits for researchers working with existing ATAC–RNA paired data but also sets the stage for the next generation of multi-omic integration algorithms.

## Supplementary Material

btaf392_Supplementary_Data

## Data Availability

All data used in this study are accessible from public sources. The data sets PBMC3k, PBMC10k, and PBMCMultiome, along with a single-nucleus data set, can be downloaded from the 10X Genomics website (https://www.10xgenomics.com/resources/datasets). The PBMCMultiome dataset can also be obtained from the ‘SeuratData’ package. The adult mouse brain SNARE-seq dataset was downloaded from GSE126074. The source codes of scMGCL are available at: (https://github.com/zlCreator/scMGCL).
