# Supplementary material for: scMGCL: accurate and efficient integration representation of single-cell multi-omics data
Source: Bioinformatics. 2025 Jul 9;41(7):btaf392. doi: 10.1093/bioinformatics/btaf392 (PMC12308176; doi:10.1093/bioinformatics/btaf392)
Supplement: btaf392_Supplementary_Data [file btaf392_supplementary_data.pdf]

Supplementary for scMGCL: accurate and efficient integration representation of single-cell multiomics data

Supplementary Notes

Data preprocess

For scATAC-seq data, we excluded low-quality cells with fewer than 1,800 reads or more than 100,000 reads. For scRNA-seq data, cells with fewer than 1,000 unique molecular identifiers (UMIs) or more than 25,000 UMIs were removed. To reduce cross-omics alignment bias due to chromatin abnormalities, we filtered out cells with nucleosome signal scores > 2 (indicating aberrant chromatin structure) or transcription start site (TSS) enrichment scores ≤ 1 (suggesting poor accessibility). For the SNARE-seq dataset, we additionally excluded cells with blacklist fraction ≥ 0.03, TSS enrichment ≥ 20, ≤ 800 RNA UMIs, or ≤ 500 ATAC reads. The processed dataset is as follows, with relevant details provided in Table S1:

- **PBMC3k** contains approximately 3, 000 cells with paired RNA and ATAC data. It serves as a foundational dataset to demonstrate scMGCL's integration capability.
- **PBMC10k** is an extended version of the PBMC3k dataset, it includes over 10, 000 cells with higher data dimensionality. This dataset allows us to explore a broader range of cell types and states while also testing the computational efficiency and scalability of our method.
- **PBMCMultiome** comprises exactly 10,412 cells but with even higher dimensional features per cell, published in 2020, further validating the scalability of scMGCL.
- **SNARE-seq** includes gene expression data from cells isolated from the mouse brain and is used to assess the efficiency of scMGCL in integrating data across different cellular components.

Then, we normalized both the RNA and ATAC data ($scanpy.pp.normalize\_total$) and applied a log transformation ($scanpy.pp.log1p$), both using default parameters. We selected the top 2000 highly variable genes ($scanpy.pp.highlyvariable\_genes$) and standardized the data ($scanpy.pp.scale$).

Baseline methods

- **MultiMAP (v1.0)**: Integrates multi-moics data via joint dimensionality reduction and optimal transport-based mapping. It aligns modalities by computing Wasserstein distances, enabling accurate cross-omics cell matching in a shared low-dimensional space.
- **SCALEX (v1.0.2)**: Applies a decoupled learning strategy to separate biological signals from batch effects. By embedding multi-moics data into a common latent space, it achieves biologically faithful integration while eliminating technical noise.

- **Scanorama (v1.7.4)**: Uses a mutual nearest neighbors (MNN) strategy to match biologically similar cells across batches, followed by singular value decomposition (SVD)-based correction. This preserves global structure and removes batch effects without relying on predefined labels.

- **scCorrector**: Combines generative adversarial networks (GANs) and contrastive learning for accurate modality alignment. GANs remove technical noise, while contrastive learning preserves biological distinctions, yielding robust integration with minimal loss of heterogeneity.

- **uniPort (v1.2.2)**: Integrates GANs and variational autoencoders (VAEs) to learn modality-invariant features in a shared latent space. This dual architecture enables both batch correction and preservation of biological variability, without requiring paired samples.

- **Harmony (v1.0)**: Performs data integration via iterative soft clustering and linear model adjustment. It maximizes alignment across datasets while preserving cell-type-specific variation.

- **Seurat (v5.1.0)**: A widely used single-cell analysis toolkit that performs integration through reciprocal anchor-based graph alignment. It combines canonical correlation analysis (CCA) and mutual nearest neighbors (MNNs) to match and align datasets while maintaining biological fidelity.

To ensure transparency and fairness in the benchmarking, default parameters were used for uniPort, Scanorama, Seurat, Harmony, and MultiMap during model training. For scCorrector, we set $preprocessed$ = True (while keeping other parameters default) since input data underwent standardized preprocessing. For Scalex, we adjusted $min\_features$ = 200 (from the default 600) during preprocessing to accommodate high-throughput datasets, consistent with established practices for such data.

Integration performance metrics

Performance metrics included: 1) Metrics for validating integration accuracy against known labels: Normalized Mutual Information (NMI), Adjusted Rand Index (ARI), F1 Score ($F1$), the sum of the above three metrics (SUM); 2) Structure-focused metrics for integration quality: Batch Entropy (BE) (modality mixing) and Silhouette Coefficient (SI) (cluster coherence).

- **Adjusted Rand Index** The Adjusted Rand Index (ARI) is a measure of the similarity between two clusterings, adjusted for chance. It ranges from -1 to 1, where 1 indicates perfect agreement, 0 indicates random labeling and negative values indicate worse-than-random agreement.

$$ARI = \frac{RI - ExpectedRI}{\max(RI) - ExpectedRI} \tag{1}$$

  where RI is the Rand Index, which measures the similarity between the clustering result and the ground truth labels. $ExpectedRI$ is the expected Rand Index under random label assignment.

- **Normalized Mutual Information** Normalized Mutual Information (NMI) quantifies the agreement between two clusterings, normalized to account for the number of clusters. It ranges from 0 to 1, where 1 indicates perfect agreement.

$$NMI = \frac{2 \cdot I(X, Y)}{H(X) + H(Y)} \tag{2}$$

  where $I(X, Y)$ is the mutual information between the clustering result X and the ground truth labels Y, while $H(X)$ and $H(Y)$ are the entropies of X and Y, respectively.

- **F1 Score** The $F1$ Score is the harmonic mean of precision and recall, commonly used to evaluate classification models, especially in cases of class imbalance. It ranges from 0 to 1, where 1 indicates perfect precision and recall.

$$F1 = \frac{2 \cdot TP}{2 \cdot TP + FP + FN} \tag{3}$$

where TP (True Positives) are the correctly predicted positive samples, FP (False Positives) are the incorrectly predicted positive samples, and FN (False Negatives) are the positive samples incorrectly predicted as negative.

- **SUM** We define SUM as the sum of ARI, NMI, and $F1$ to comprehensively assess integration performance. Each metric offers a distinct yet complementary perspective: ARI captures cluster assignment consistency, NMI assesses information overlap, and $F1$ reflects classification quality. Combining them into a single SUM score allows for a robust and multidimensional evaluation of integration accuracy. This composite metric has also been adopted in prior multi-omics integration studies (e.g., uniPort), providing precedent and validation for its use.

$$SUM = ARI + NMI + F1 \tag{4}$$

- **Batch Entropy** We computed the Batch Entropy (BE) score to quantify the degree of mixing between cells from different datasets by evaluating the regional mixing entropies at the locations of randomly selected cells. A higher score indicates better integration and mixing of cells across datasets. The Batch Entropy score is computed using the following equations:

$$p_i^r = \frac{p_i / P_i}{\sum_{t=1}^{n} p_i / P_i}, \tag{5}$$

$$BE = -\sum_{t=1}^{n} p_i^r \log(p_i^r), \tag{6}$$

where p_i represents the proportion of cells from batch i in a specific region, and P_i denotes the proportion of the total cell count that belongs to batch i.

- **Silhouette coefficient** The Silhouette Coefficient (SI) measures the compactness and separation of clusters. Its maximum value is 1, and the larger the value, the better the performance in distinguishing different clusters.

$$SI = \frac{b - a}{\max(a, b)} \tag{7}$$

where a is the average distance between a sample and all other samples in the same cluster, and b is the average distance between a sample and all samples in the nearest neighboring cluster.

- **Mean accuracy** $Mean\ accuracy$ calculates a cell-type-weighted mean accuracy to evaluate classification performance across imbalanced cell types.

$$Mean\ accuracy = \frac{\sum_{i=t_1}^{t_n} w_i \cdot ac_i}{\sum_{i=t_1}^{t_n} w_i} \tag{8}$$

for each cell type i, we compute its class-specific accuracy ac_i, then weigh it by the number of cells w_i belonging to that type.

- **Marker-gene correlation** The Marker-gene correlation (r) calculates a weighted Pearson correlation coefficient across cell types to quantify the concordance between RNA and ATAC modalities at the gene level.

$$r = \frac{\sum_{i=t_1}^{t_n} w_i(x_i - \bar{x}_w)(y_i - \bar{y}_w)}{\sqrt{\sum_{i=t_1}^{t_n} w_i(x_i - \bar{x}_w)^2}\sqrt{\sum_{i=t_1}^{t_n} w_i(y_i - \bar{y}_w)^2}} \tag{9}$$

  for each cell type i, we computed the mean expression of the gene in RNA (x_i) and ATAC (y_i) and used the number of cells in RNA (w_i) as weights.

Marker gene analysis across datasets

In the PBMC datasets (Figures 5B, S9, and S10), expression patterns of well-known marker genes are consistent with known cell-type identities. $TREM1$, a key marker of myeloid cell activation involved in pro-inflammatory responses, is highly expressed in macrophages. $NKG7$, a effector molecule, is predominantly expressed in NK cells and CD8+ T cells, reflecting their role in target cell killing. LYZ (Lysozyme), which aids in bacterial lysis, is strongly expressed in macrophages. $MS4A1$ (CD20), essential for B cell development and activation, shows specific and high expression in B cells.

In the SNARE-seq (Figure S11), marker gene expression reflects distinct neuronal subtypes. $Fezf2$, a transcription factor that defines neuronal subtype identity, is highly enriched in layer 5 subcerebral projection neurons (SCPNs) of the cortex. $SYT6$ (Synaptotagmin-6), involved in synaptic vesicle regulation, is broadly expressed but shows peak expression in layer 6. $TRF1$ (Transferrin Receptor 1), essential for oligodendrocyte function and iron homeostasis, is enriched in oligodendrocytes. SST (Somatostatin), an inhibitory neuromodulator, is highly expressed in SST+ interneurons across multiple cortical layers, where it contributes to neural circuit regulation.

Supplementary Ablation studies

Clustering methods The KNN-based method achieved the shortest processing time (see “KNN” in Table S4) while maintaining effective and consistent alignment across integrated datasets (Figure S18). In contrast, the graph Laplacian approach incurred significantly higher computational costs ($\triangle time >$ 6.31 seconds vs. KNN, Table S4) due to its dependence on global structure information, and its performance is degraded in the presence of cellular heterogeneity — for example, splitting CD8 TEM1 into two separate clusters in the PBMCMultiome dataset (Figure S18). Clustering-based methods also suffer from requiring pre-specified cluster numbers, making them less suitable for unlabeled data. The SNN method had the longest processing time across datasets (see “SNN” in Table S4) and occasionally overfit heterogeneous regions, misinterpreting noise as biological signals and generating unstable, non-generalizable clusters (e.g., PBMC3k, Figure S18). Based on its computational efficiency and robustness across datasets, we selected the KNN-based method as our default clustering strategy.

Similarity matrix construction methods While both cosine similarity and Pearson correlation yielded reasonable integration results, they introduced subtle misalignment artifacts — for example, partial separation of CD14+ monocytes in the PBMC3k dataset (Figure S19). Additionally, in the SNARE-seq dataset, both metrics exhibited overfitting tendencies, as reflected by the elongated, streak-like distribution of astrocyte cells. In contrast, Euclidean distance consistently produced more compact and biologically coherent clusters across datasets. Based on these observations, we selected Euclidean distance as the default metric in our graph construction step due to its superior integration stability and robustness.

Augmentation methods To compare, we applied independent augmentations to RNA and ATAC modalities using the same model. In PBMC3k (Figure S20), this resulted in chaotic modality overlap, dispersed cell-type distributions, and poor cluster resolution — indicating severe biological signal loss.

We also tested cross-modal collaborative augmentation, where both modalities received equal perturbation intensity during training. While this approach improved cluster compactness relative to independent augmentation (Figure S21), it failed to fully resolve inter-cluster heterogeneity and did not achieve tight modality alignment. In contrast, treating modalities as mutual augmentations yielded well-separated, biologically meaningful clusters and complete modality alignment (e.g., scMGCL on PBMC10k, Figure 3), demonstrating the effectiveness and feasibility of our approach.

Supplementary Tables and Figures

Dataset	Cells	Genes	Peaks	Cell Types
PBMC3k	2780	19049	81156	22
PBMC10k	11290	25286	111857	30
PBMCMultiome	10412	36601	108377	19
SNARE-seq	9134	16750	241757	10

Table S1: Statistics of the datasets.

Dataset	Gene	Correlation	
		After integration	Before integration
PBMC3k	$LEF1$	0.988	0.984
	$CD3D$	0.980	0.965
	$MS4A1$	0.989	0.988
	$NKG7$	0.944	0.932
	LYZ	0.958	0.946
	$TREM1$	0.927	0.941
PBMC10k	$LEF1$	0.988	0.989
	$CD3D$	0.976	0.979
	$MS4A1$	0.989	0.990
	$NKG7$	0.935	0.938
	LYZ	0.899	0.934
	$TREM1$	0.931	0.940
PBMCMultiome	$LEF1$	0.989	0.987
	$CD3D$	0.972	0.972
	$MS4A1$	0.974	0.971
	$NKG7$	0.945	0.949
	LYZ	0.986	0.987
	$TREM1$	0.947	0.949
SNARE-seq	$Aqp4$	0.878	0.896
	$Slc17a7$	0.935	0.933
	$Fezf2$	0.970	0.957
	$Syt6$	0.956	0.960
	Trf	0.945	0.967
	sst	0.924	0.888

Table S2: Summary of marker-gene correlations

Method	GPU/CPU	Accelerator	Memory	Parallelization	Computing Unit
scMGCL	GPU	NVIDIA RTX 4090 (24GB)	120 GB DDR4 RAM	CUDA v12.1	1×GPU (exclusive)
uniPort	GPU	NVIDIA RTX 4090 (24GB)	120 GB DDR4 RAM	CUDA v12.1	1×GPU (exclusive)
Seurat	CPU	–	120 GB DDR4 RAM	OpenMP (64 threads)	32 physical cores
Harmony	CPU	–	120 GB DDR4 RAM	OpenMP (64 threads)	32 physical cores
MultiMap	CPU	–	120 GB DDR4 RAM	OpenMP (64 threads)	32 physical cores
Scalex	GPU	NVIDIA RTX 4090 (24GB)	120 GB DDR4 RAM	CUDA v12.1	1×GPU (exclusive)
Scanorama	CPU	–	120 GB DDR4 RAM	OpenMP (64 threads)	32 physical cores
scCorrector	GPU	NVIDIA RTX 4090 (24GB)	120 GB DDR4 RAM	CUDA v12.1	1×GPU (exclusive)

Table S3: Summary of computational resource allocation across methods

Dataset	Method		
	Laplacian	SNN	KNN
PBMC3k	6.81	14.63	**0.5**
PBMC10k	30.13	82.43	**9.5**
PBMCMultiome	29.98	70.93	**8.46**
SNARE-seq	24.85	80.47	**7.23**

Table S4: **Processing times of clustering methods (seconds).** The shortest processing time for each dataset is highlighted in bold.

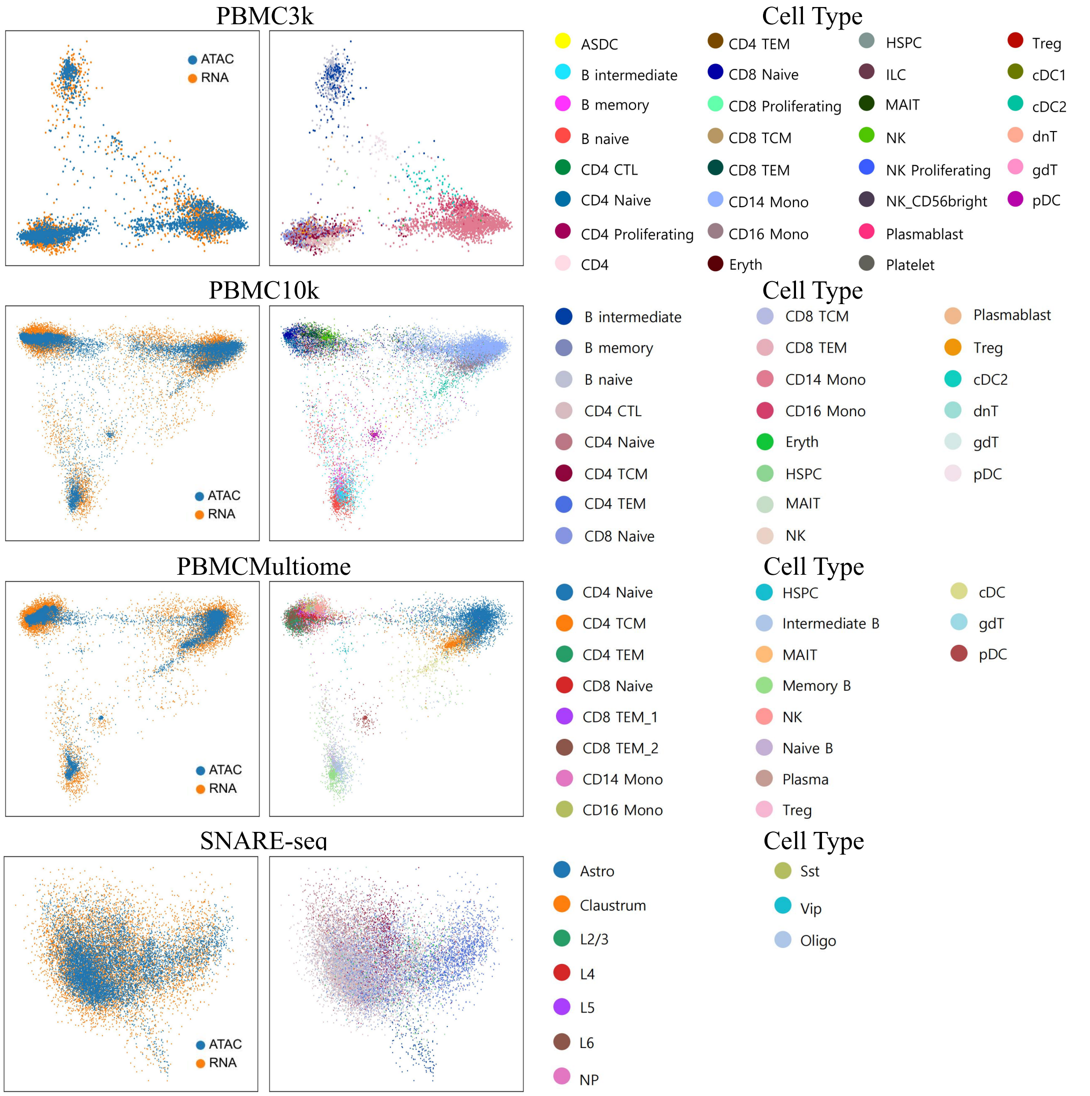

Figure S1: UMAP visualization integration results of Harmony on all datasets.

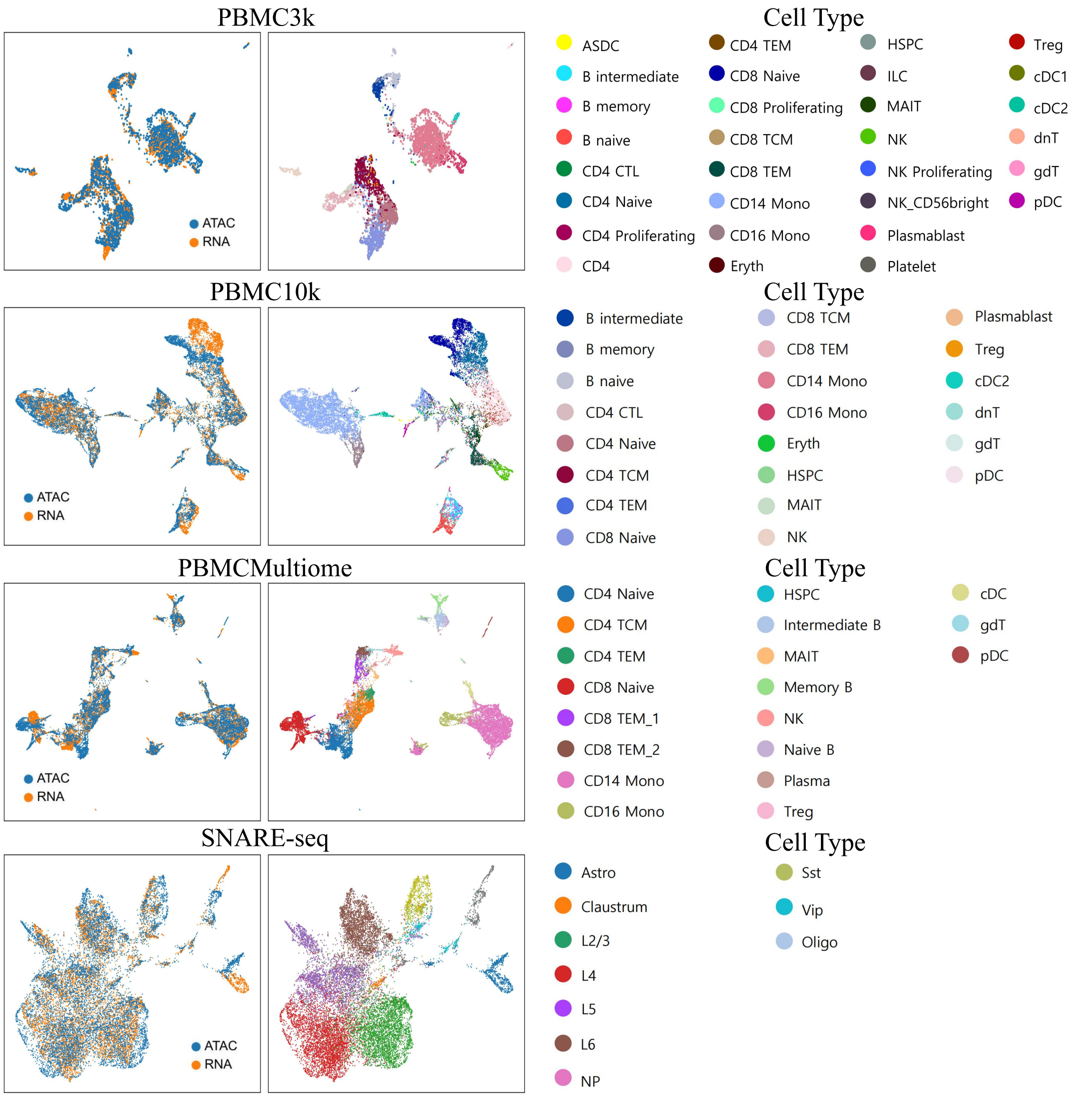

Figure S2: UMAP visualization integration results of Seurat on all datasets.

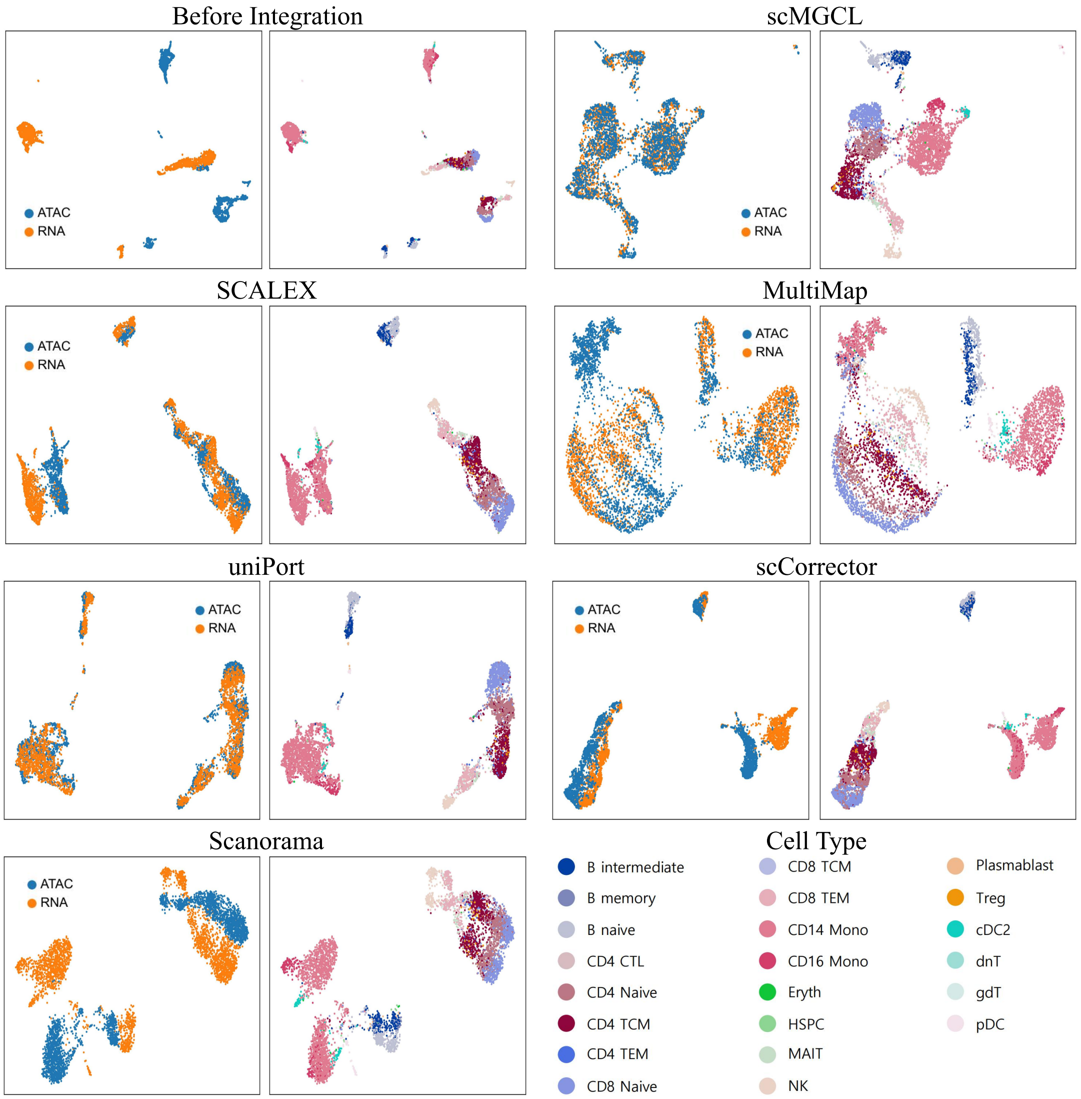

Figure S3: UMAP visualization comparing integration results of scMGCL and benchmark methods on PBMC3k dataset.

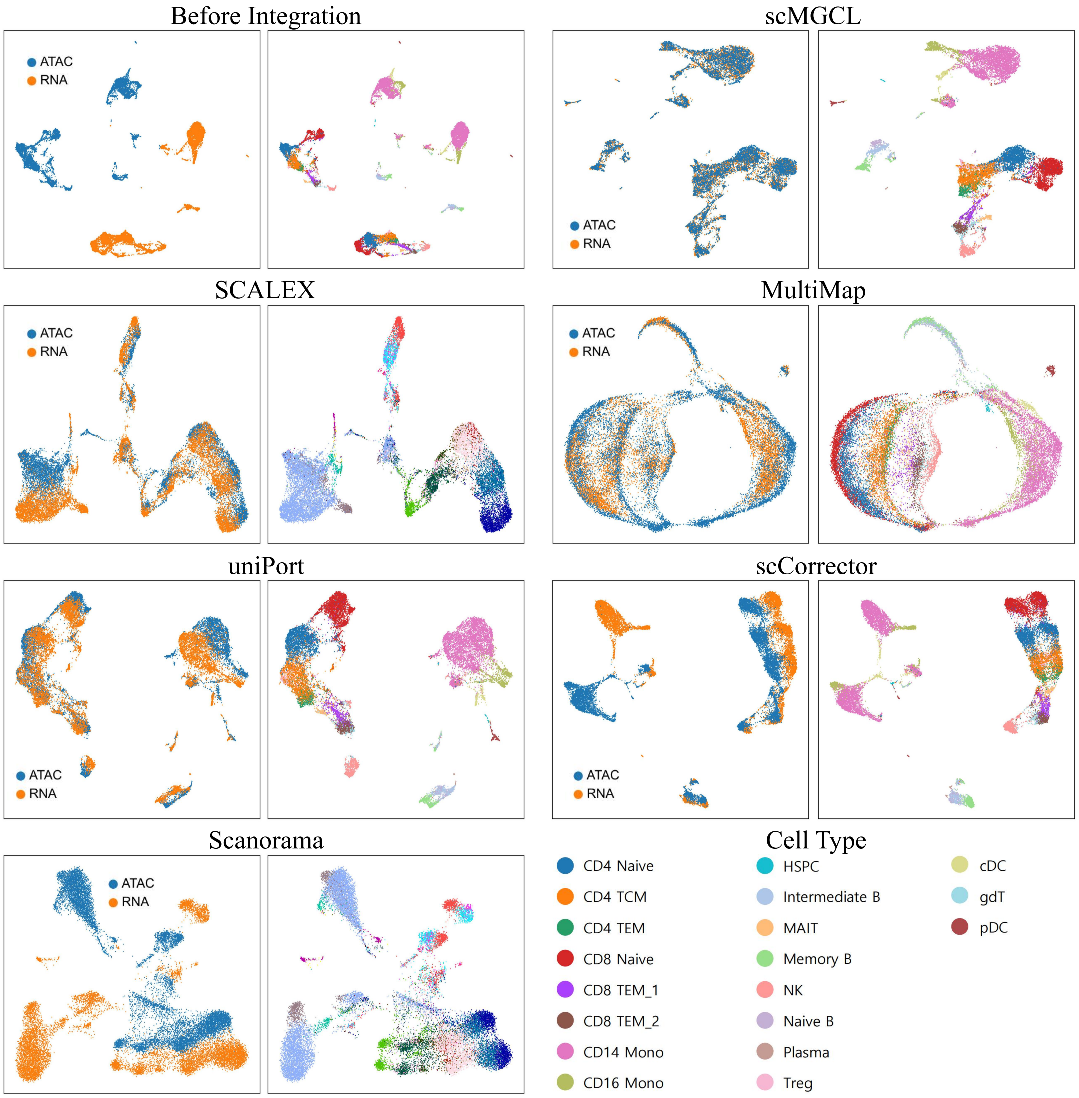

Figure S4: UMAP visualization comparing integration results of scMGCL and benchmark methods on PBMCMultiome dataset.

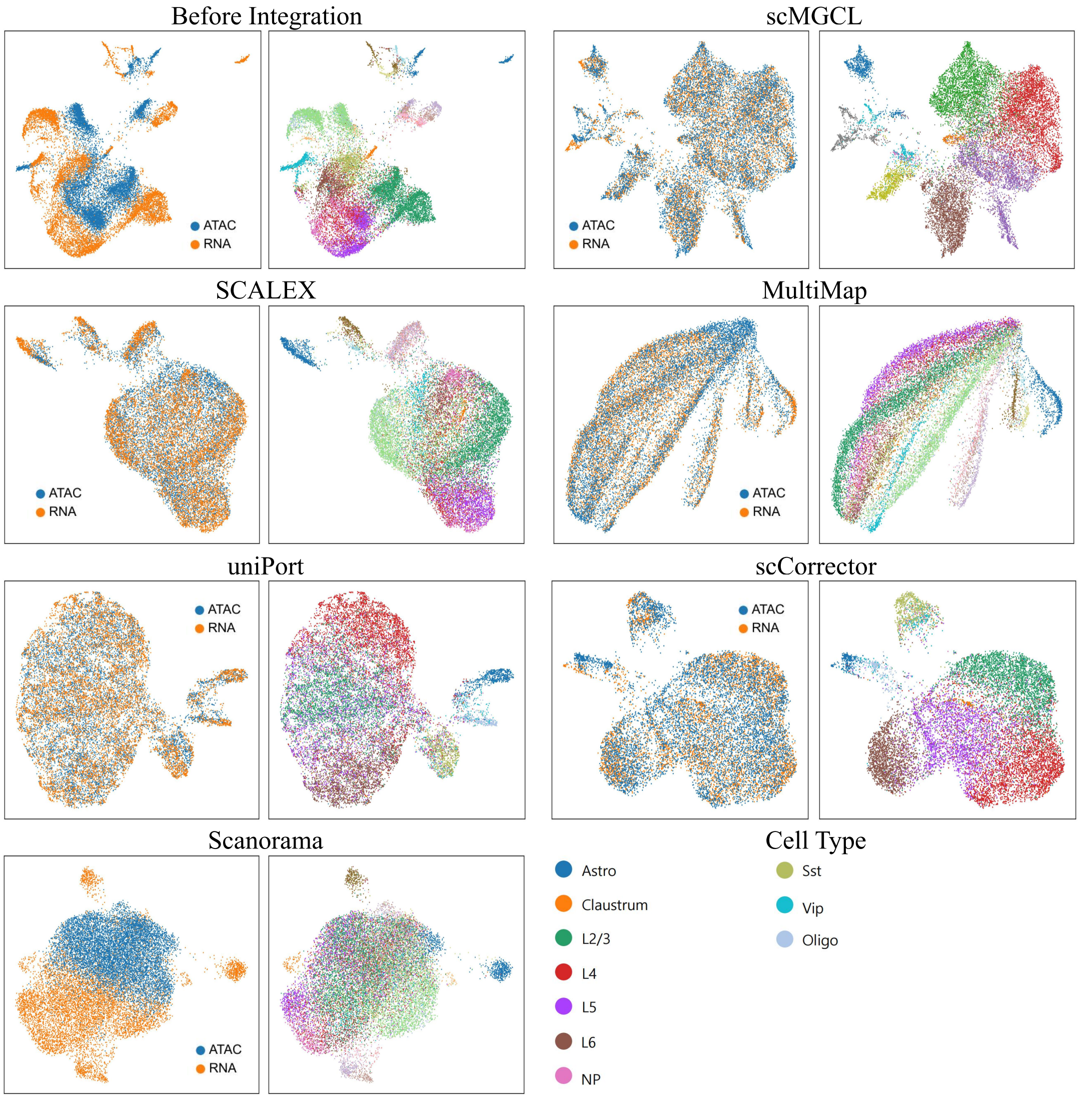

Figure S5: UMAP visualization comparing integration results of scMGCL and benchmark methods on SNARE-seq dataset.

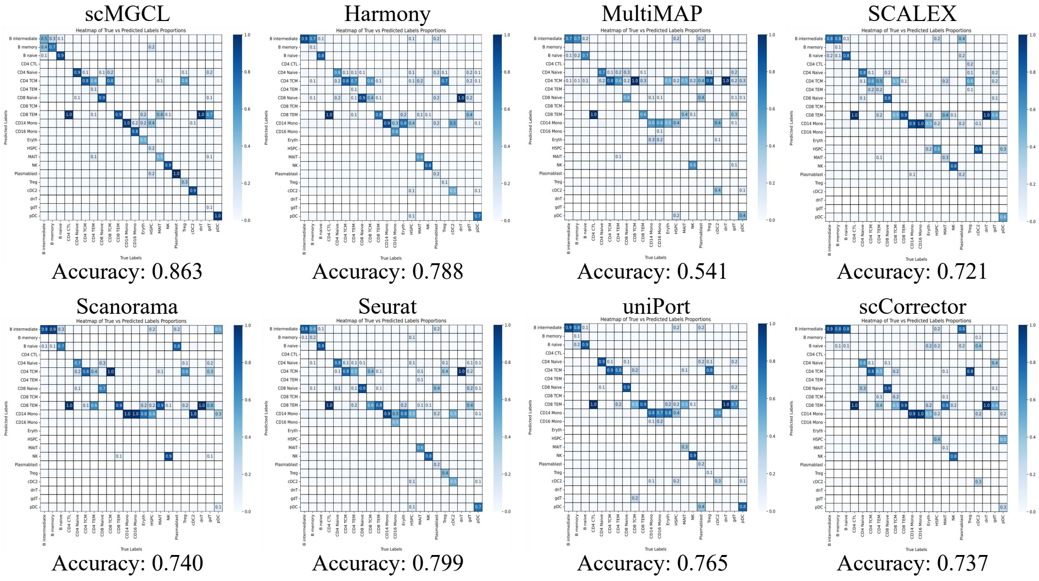

Figure S6: Confusion matrix heatmap of true cell types and predicted cell types on PBMC3k.

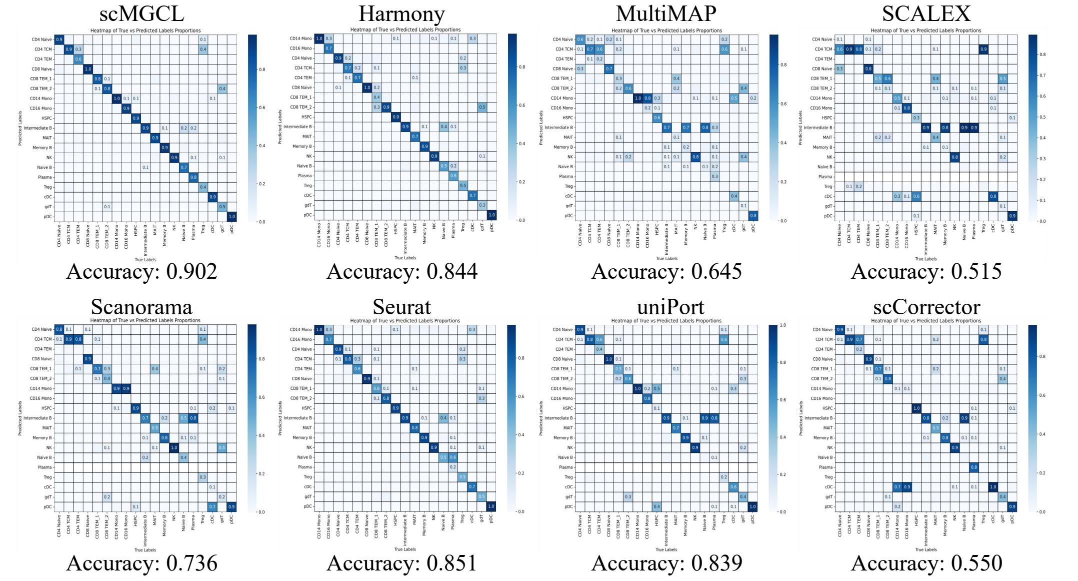

Figure S7: Confusion matrix heatmap of true cell types and predicted cell types on PBMCMultiome.

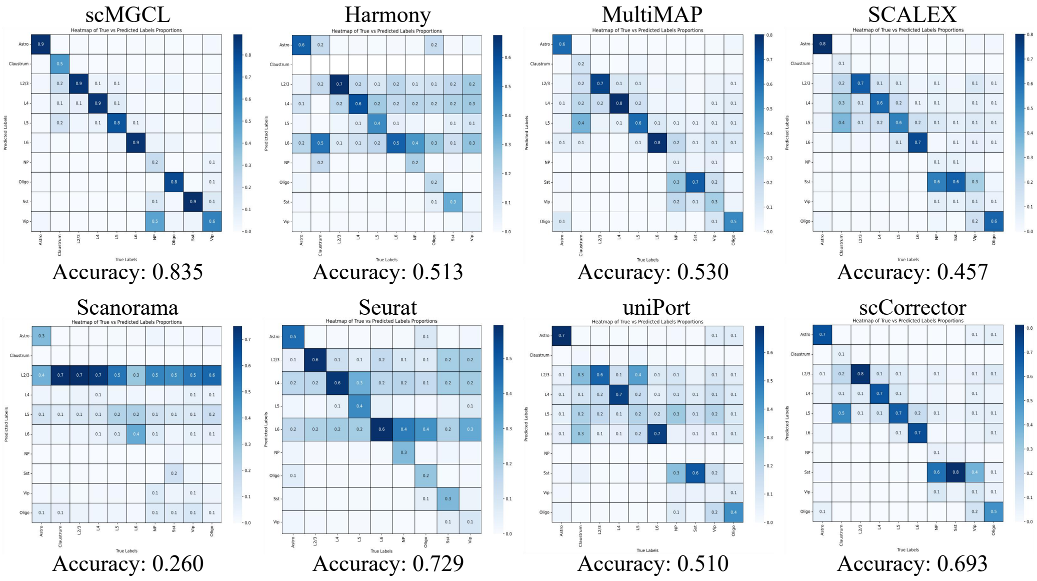

Figure S8: Confusion matrix heatmap of true cell types and predicted cell types on SNARE-seq.

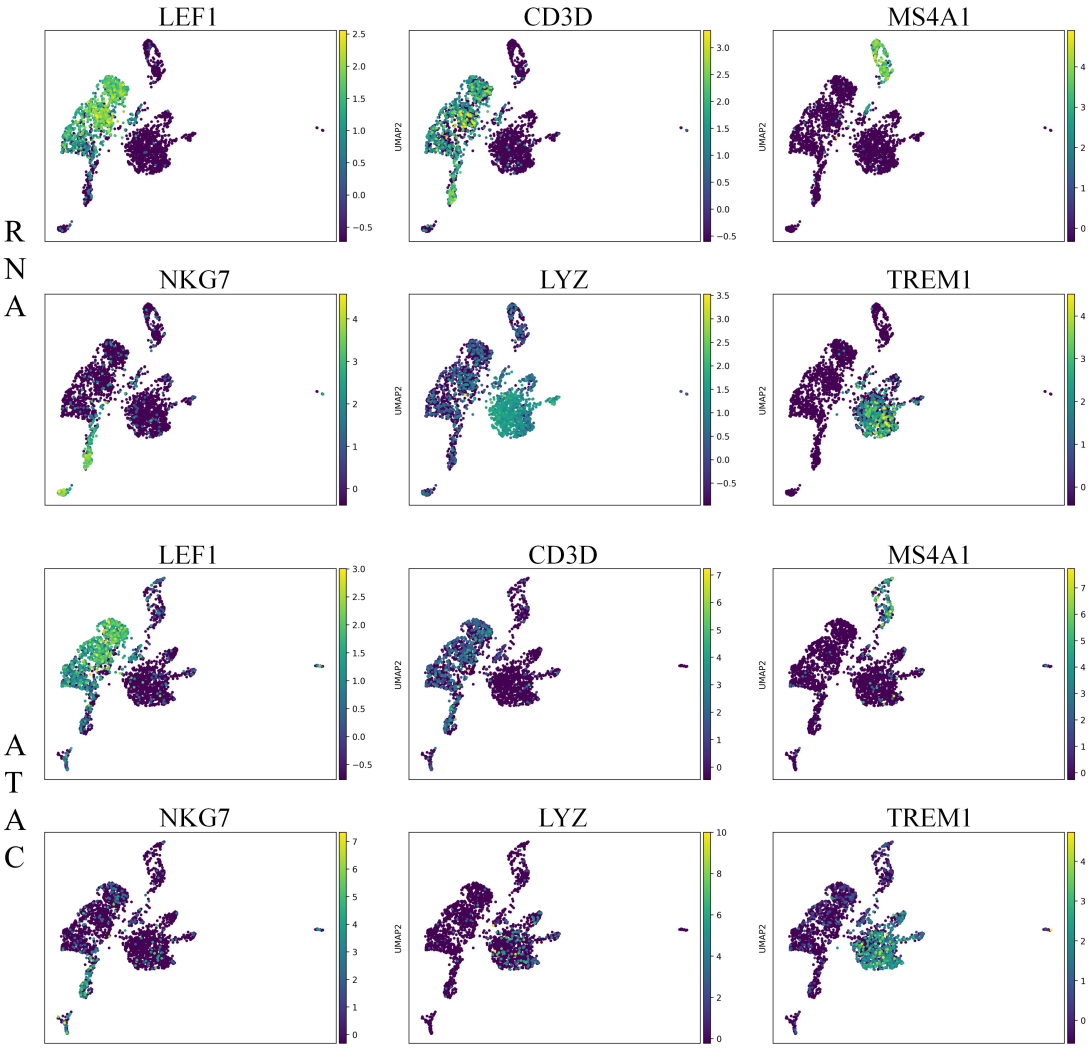

Figure S9: Marker expression for integrated ATAC cells and RNA cells in PBMC3k dataset.

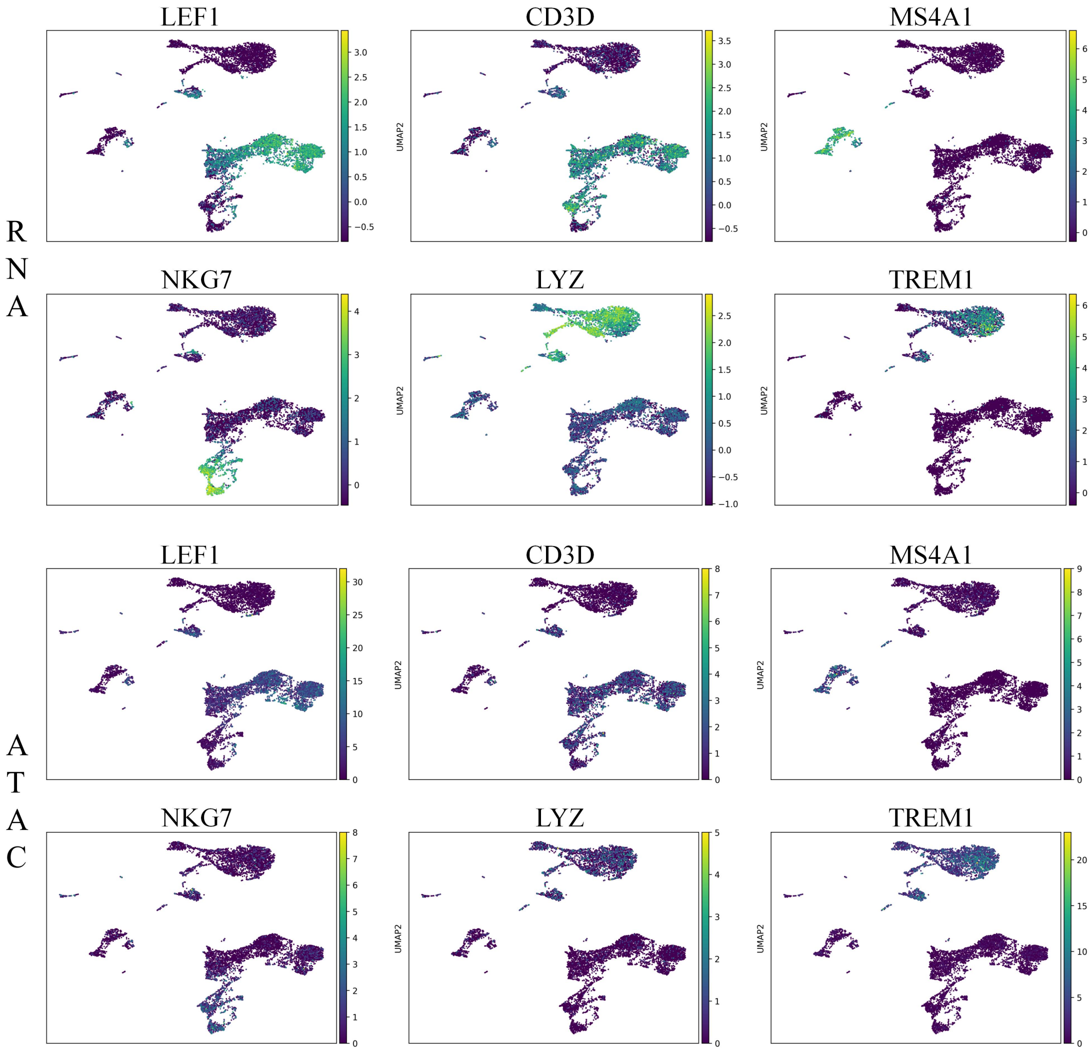

Figure S10: Marker expression for integrated ATAC cells and RNA cells in PBMCMultiome dataset.

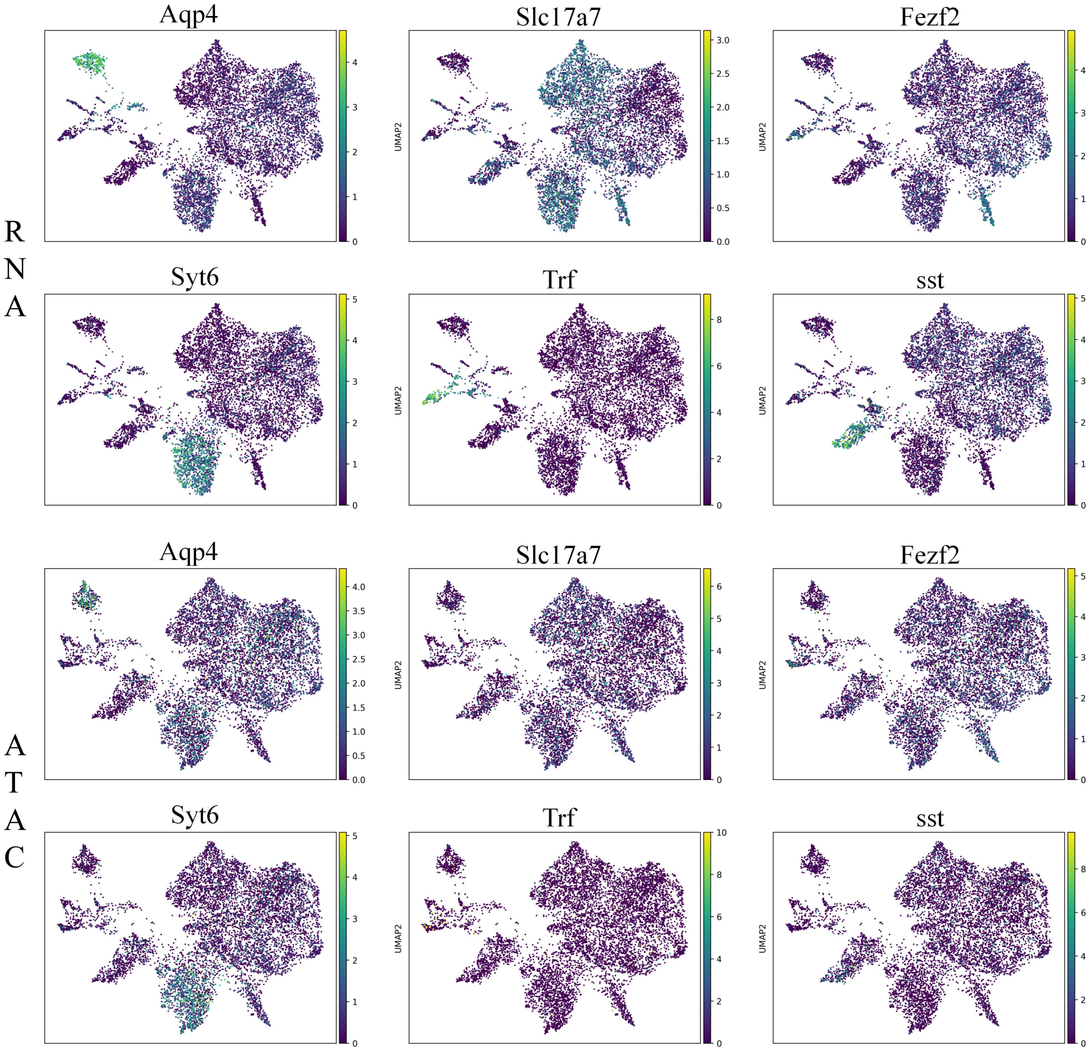

Figure S11: Marker expression for integrated ATAC cells and RNA cells in SNARE-seq dataset.

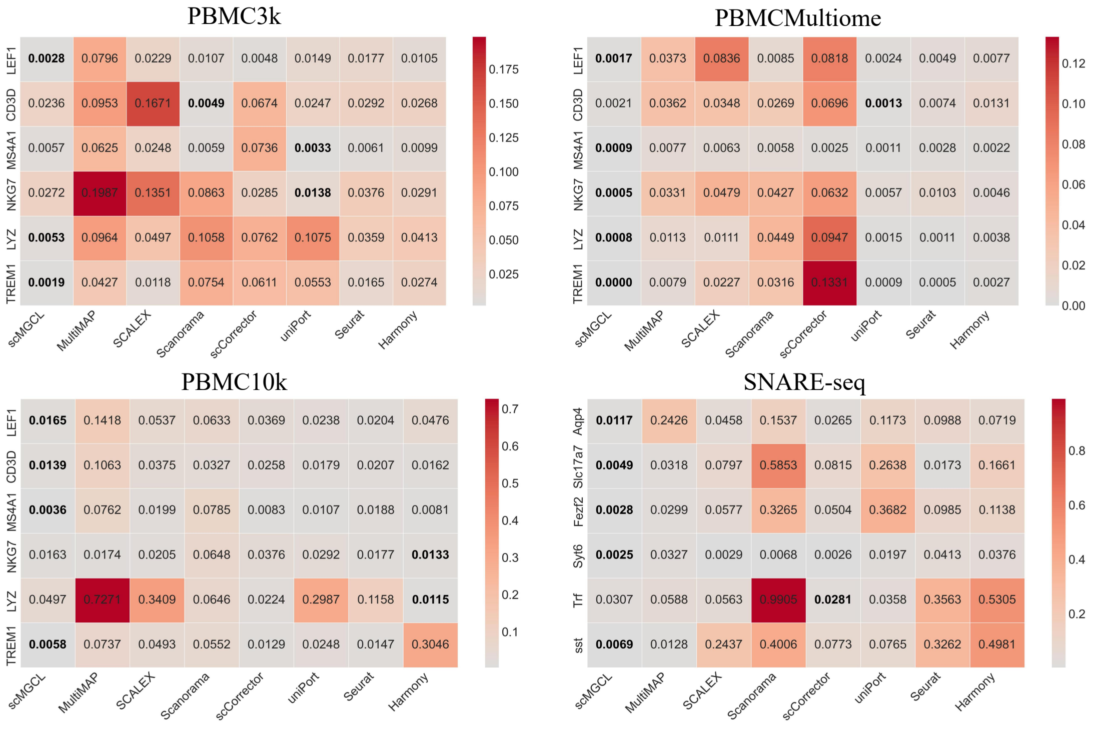

Figure S12: **Evaluation of biological signal loss (l) for scMGCL and benchmark methods across single-cell multi-omics datasets.** Biological signal loss was quantified as (1 - Pearson correlation) between marker gene accessibility in pre-integration and integrated ATAC data, representing the degree of biological information degradation. For each marker gene, the method with the lowest signal loss (i.e., highest correlation) is highlighted in bold.

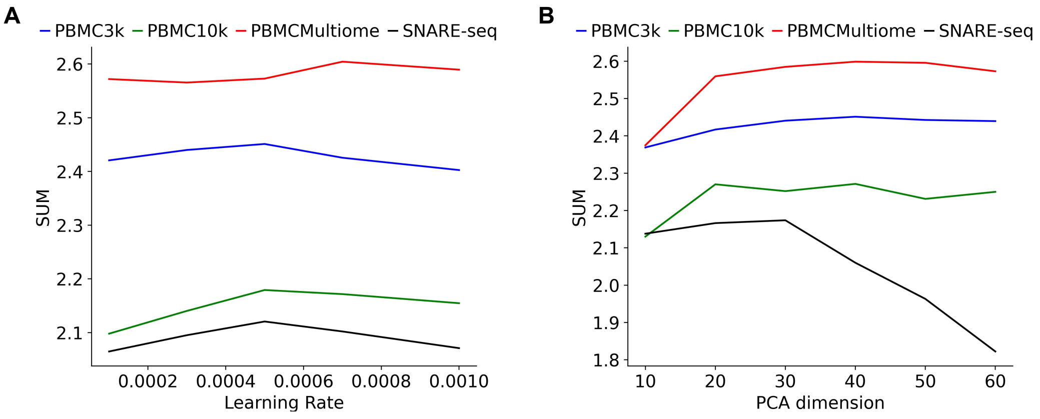

Figure 13: **scMGCL integration performance with varying parameters. (A)** scMGCL integration performance with varying learning rate across all datasets. **(B)** scMGCL integration performance with varying PCA dimension across all datasets.

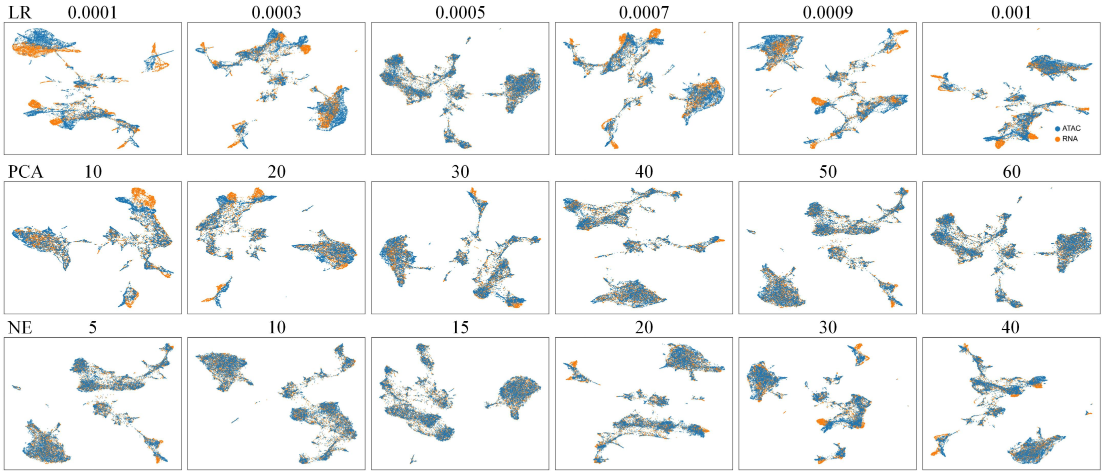

Figure S14: UMAP visualization of scMGCL integration results on PBMC10k datasets under varying Learning Rates (LR), PCA dimensions, and Numbers of Neighboring Edges (NE).

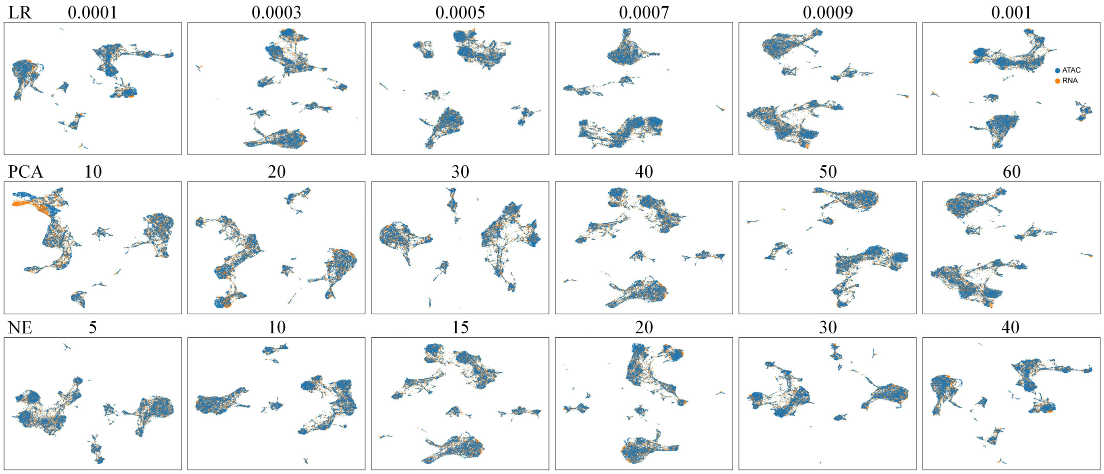

Figure S15: UMAP visualization of scMGCL integration results on PBMCMultiome datasets under varying Learning Rates (LR), PCA dimensions, and Numbers of Neighboring Edges (NE).

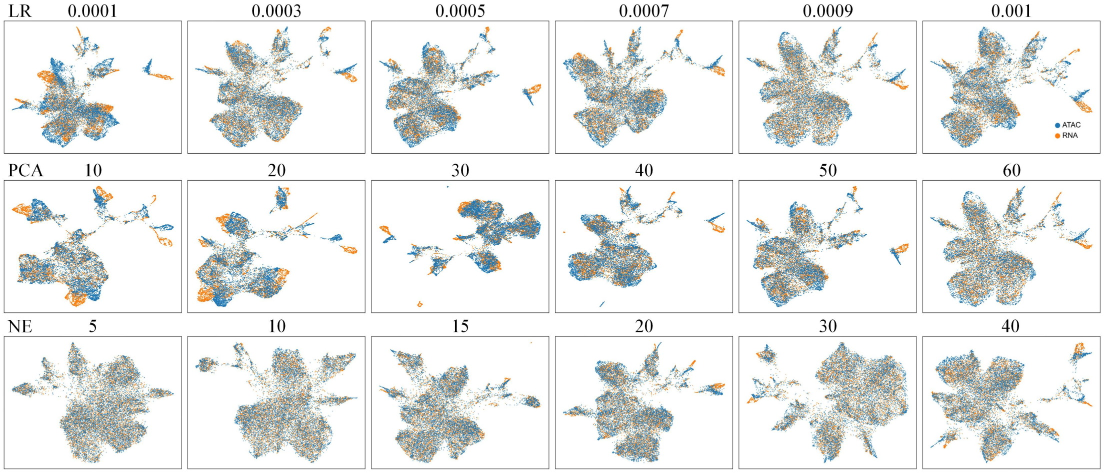

Figure S16: UMAP visualization of scMGCL integration results on SNARE-seq datasets under varying Learning Rates (LR), PCA dimensions, and Numbers of Neighboring Edges (NE).

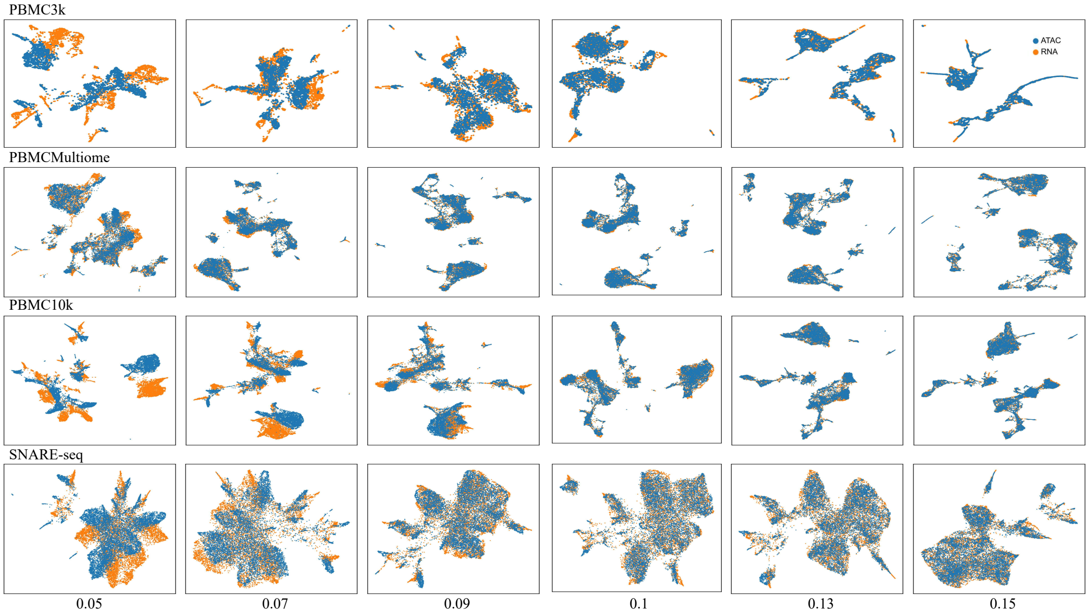

Figure S17: scMGCL integration performance with varying temperature parameter.

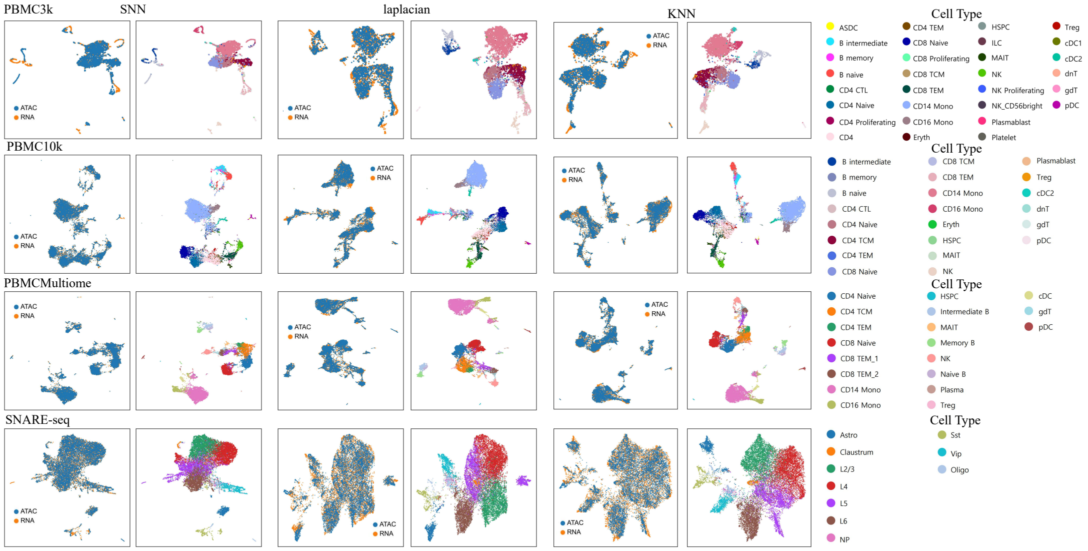

Figure S18: UMAP visualization integration results of different nearest neighbor construction methods across datasets.

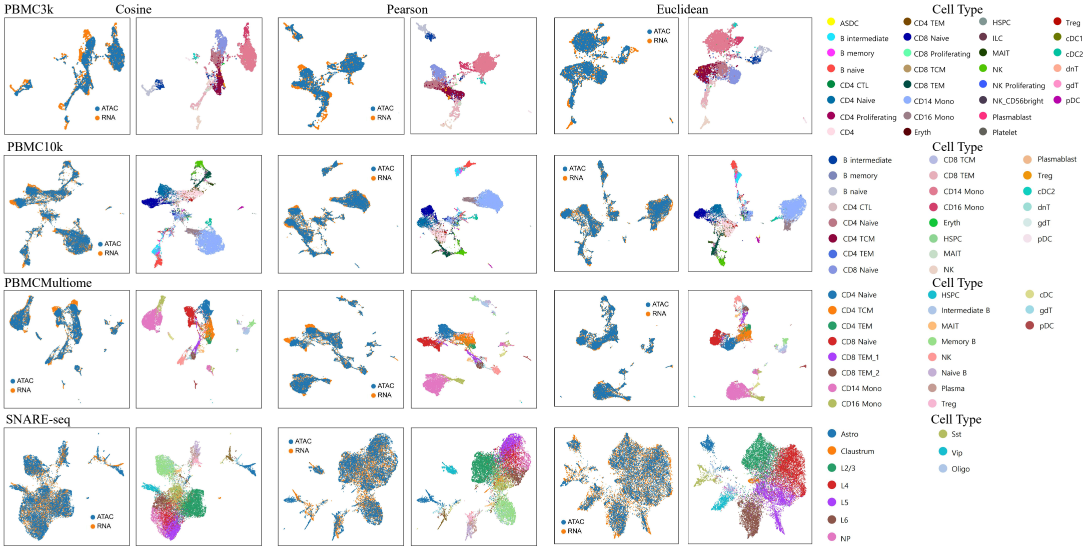

Figure S19: UMAP visualization of integration results using different similarity metrics (Euclidean distance, cosine similarity, and Pearson correlation) for cell–cell graph construction across datasets.

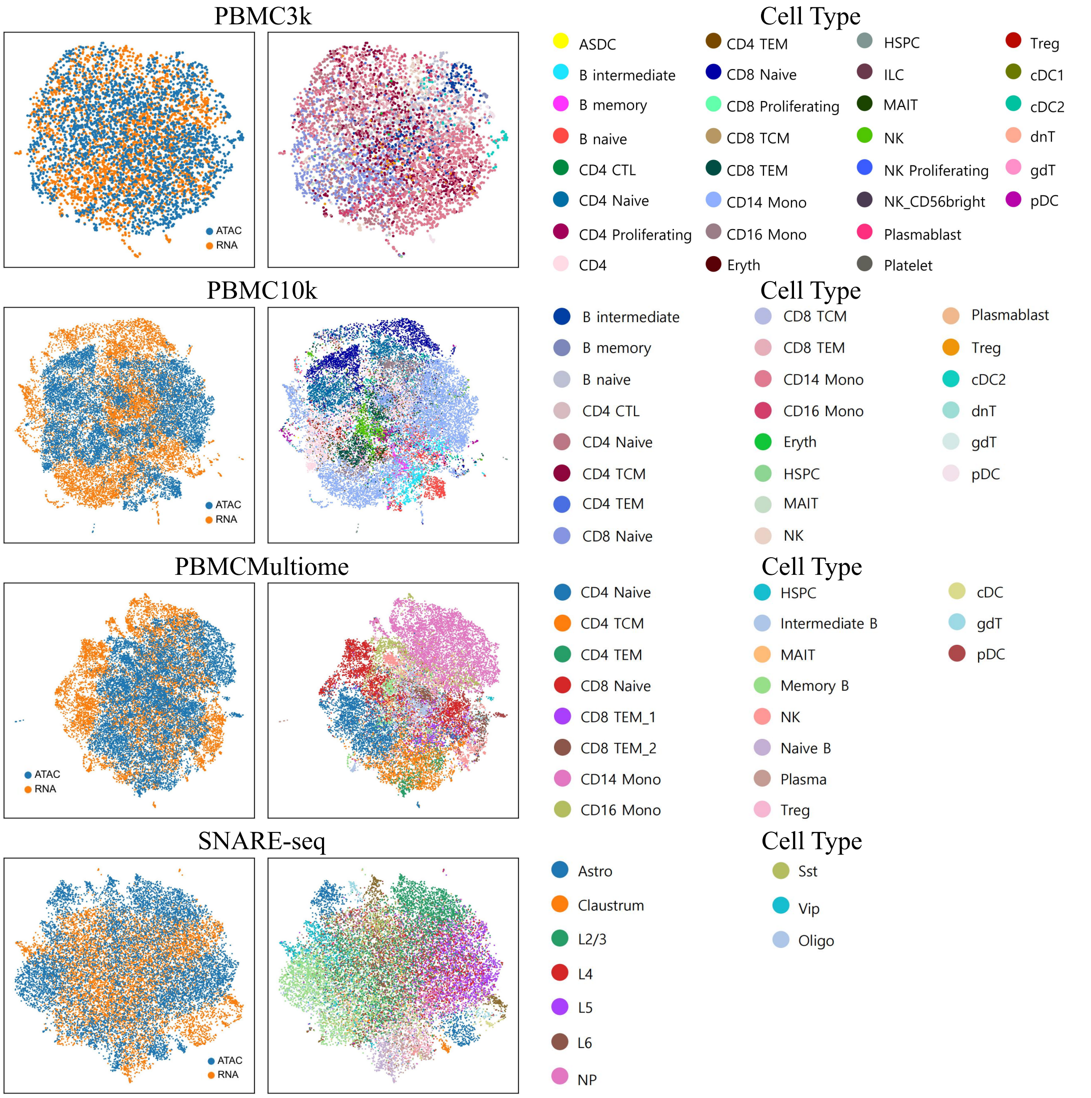

Figure S20: UMAP visualization of integration results using independent random augmentations applied separately to RNA and ATAC modalities across all datasets.

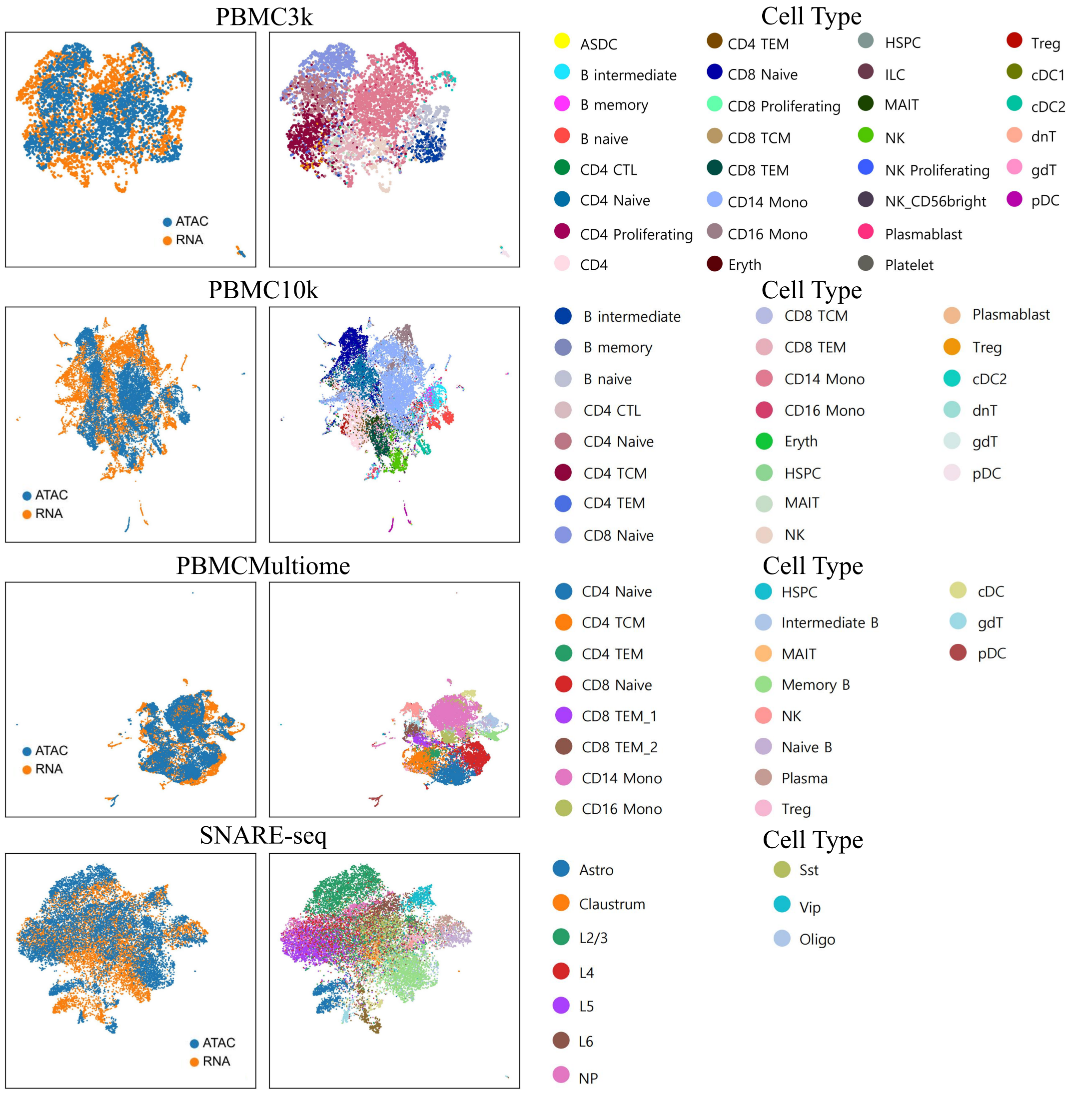

Figure S21: UMAP visualization of integration results using cross-modal collaborative augmentation across all datasets.

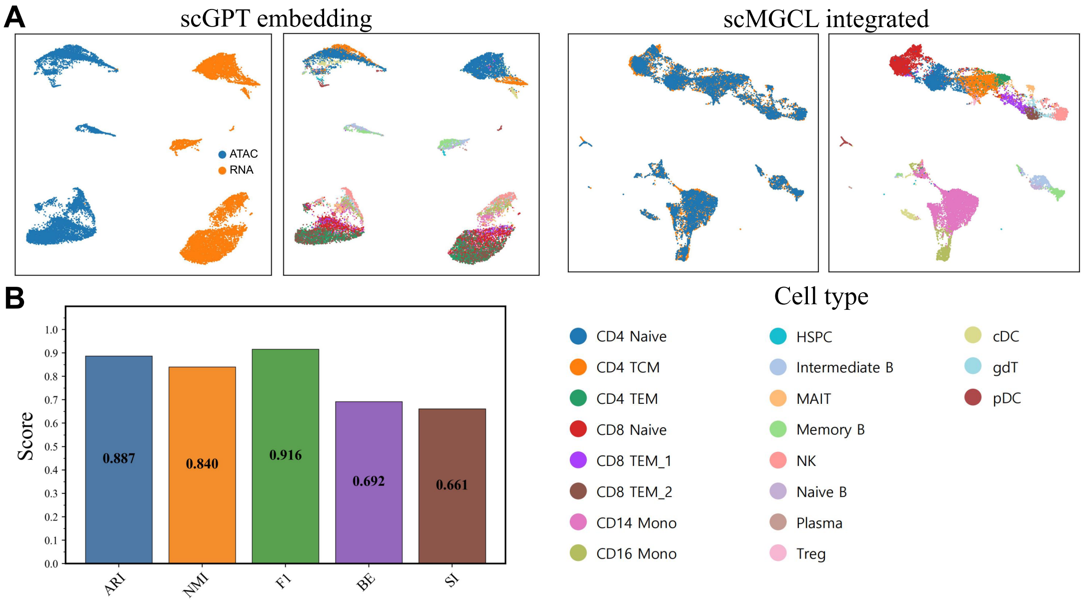

Figure S22: **Performance of scMGCL on PBMCMultiome dataset with concatenated scGPT embeddings. (A)** UMAP visualization of integration results using scMGCL with concatenated scGPT embeddings. For each subgraph, the left panels show clustering results for the individual modalities (RNA and ATAC), while the right panels display cell clusters colored by original cell type annotations. The top-left panel shows the latent representations generated by scGPT for the PBMCMultiome dataset. Cell type labels are provided in the bottom-right panel. **(B)** Evaluation metrics of scMGCL using concatenated scGPT embeddings.
